# Many-body quantum chaos and space-time translational invariance

**DOI:** 10.1038/s41467-022-34318-1

**Published:** 2022-12-05

**Authors:** Amos Chan, Saumya Shivam, David A. Huse, Andrea De Luca

**Affiliations:** 1 Princeton Center for Theoretical Science, Princeton University, Princeton, NJ 08544 USA; 2 Physics Department, Lancaster University, Lancaster, LA1 4YW USA; 3 Department of Physics, Princeton University, Princeton, NJ 08544 USA; 4 Laboratoire de Physique Théorique et Modélisation, CY Cergy Paris Université, CNRS, F-95302 Cergy-Pontoise, France

**Keywords:** Theoretical physics, Statistical physics

## Abstract

We study the consequences of having translational invariance in space and time in many-body quantum chaotic systems. We consider ensembles of random quantum circuits as minimal models of translational invariant many-body quantum chaotic systems. We evaluate the spectral form factor as a sum over many-body Feynman diagrams in the limit of large local Hilbert space dimension *q*. At sufficiently large *t*, diagrams corresponding to rigid translations dominate, reproducing the random matrix theory (RMT) behaviour. At finite *t*, we show that translational invariance introduces additional mechanisms via two novel Feynman diagrams which delay the emergence of RMT. Our analytics suggests the existence of exact scaling forms which describe the approach to RMT behavior in the scaling limit where both *t* and *L* are large while the ratio between *L* and *L*_Th_(*t*), the many-body Thouless length, is fixed. We numerically demonstrate, with simulations of two distinct circuit models, that the resulting scaling functions are universal in the scaling limit.

## Abstract (web summary)

Getting a grip on the chaotic properties of quantum systems is difficult. Now, the effect of translational invariance in space in time in an ensemble of random quantum circuits is shown to lead to largely universal scaling laws describing the system without the need of knowing microscopic details.

## Introduction

Understanding the chaotic properties of quantum systems is a notoriously hard problem. A fruitful direction has been opened by the combination of two ingredients: First, fingerprints of an underlying chaotic dynamics are visible in the Hamiltonian spectrum of quantum systems^1^; second, spectral properties are best discussed in statistical terms^2^. This approach eliminates dependence on the microscopic details of the studied systems and brings out the universal characteristics of an ensemble of statistically similar Hamiltonians, which are captured by the random matrix theory (RMT) contrained only by symmetries^3,4^. RMT provides a prototype of thermalising dynamics for which the eigenstate thermalization hypothesis^5–7^ is confirmed^8^.

**Fig. 1:**
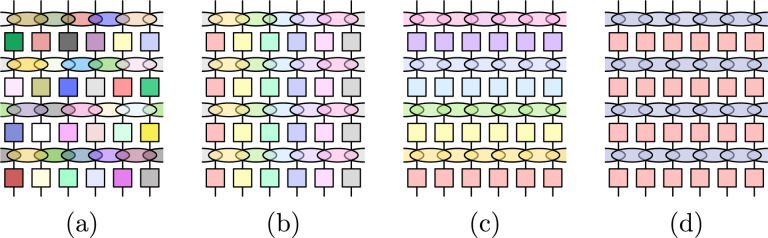
Illustrations of the different types of RUC for the random phase model. **a** Temporally and spatially random RPM; **b** Floquet (and spatially random) RPM; **c** TI (and temporally random) RPM, and **d** TI Floquet RPM. For each case, gates of the same colors are identical.

However, RMT fails to reproduce the local structure of interactions of many-body quantum systems which results in a complex geometry and correlation in the Fock space^9–14^. For this reason, random unitary circuits (RUC) have been proposed as toy models which utilize RMT while incorporating a notion of locality and dimensionality. In the simplest formulation, time evolution of RUC is performed by acting with randomly generated unitary gates on pairs of nearest neighbors in a spin lattice (Fig. 1a)^15,16^. These models have proven fruitful in developing a unifying picture of the out-of-equilibrium dynamics of generic many-body systems with predictions for the entanglement growth^15,17–25^, and the out-of-time-ordered correlators^16,26–28^. More recently, Floquet random unitary circuits (FRUC) have been introduced by applying repeatedly the same set of random gates (Fig. 1b)^29–34^. FRUC have given access to the study of non-trivial spectral properties in extended many-body systems. In particular, for the spectral form factor (SFF)^29,30,32–49^,

1 $$K(t,L)\equiv \langle {{{{{{{\rm{Tr}}}}}}}}[W(t)]{{{{{{{\rm{Tr}}}}}}}}[{W}^{{{{\dagger}}} }(t)]\rangle$$

where *W*(*t*) is the time evolution operator for time *t*, *L* is the system size and 〈…〉 indicates the ensemble average, it has been argued that the RMT behavior is recovered only for *t* > *t*_Th_(*L*), with *t*_Th_(*L*) the SFF Thouless time. *t*_Th_(*L*) is an intrinsic time scale that generally grows unbounded with the system size *L* (with the exception of the dual-unitary circuits^36–38,50^). Its origin traces back to the existence of domain walls separating growing chaotic subregions^30,51,52^.

## Results

In this article, we consider the effect of translational invariance in space and time on the SFF. We introduce a spatially translational invariant (TI) version of the *random phase model* (RPM)^30^ on a *d*–dimensional lattice of length *L*, which can also be time-periodic (Floquet) or not. We will refer to the four setups resulting from the combination of TI and time-periodicity as cases (a), (b), (c) and (d) as illustrated in Fig. 1. We show that the SFF is exactly computable in the limit of large local Hilbert space dimension *q* via a diagrammatic expansion made up of contractions between ${{{{{{{\rm{Tr}}}}}}}}[W(t)]$ and ${{{{{{{\rm{Tr}}}}}}}}[{W}^{{{{\dagger}}} }(t)]$ (respectively top and bottom layer in Fig. 3b below) of (1). Before providing the explicit derivation, we outline the main results. At large *t* ≫ *t*_Th_(*L*) (but still with *t* ≪ *t*_Heis_(*L*), the Heisenberg time which is exponentially large in the system size), only *ladder diagrams*, corresponding to rigid translations of the top layer ${{{{{{{\rm{Tr}}}}}}}}[W(t)]$ w.r.t. the bottom layer ${{{{{{{\rm{Tr}}}}}}}}[{W}^{{{{\dagger}}} }(t)]$, contribute (see Fig. 3 (a–c) below). This reproduces exactly the RMT predictions, i.e. *K*(*t*, *L*) ~ *K*_RMT_(*t*, *L*), with

2 $${K}_{{{{{{\rm{RMT}}}}}}}(t,\, L)\equiv {\left \{ \begin{array}{ll}1,\hfill&{{{{{\rm{w}}}}}}/{{{{{{{\rm{o}}}}}}}}\,{{{{{{{\rm{symm}}}}}}}}. - ({{{{{{{\rm{a}}}}}}}})\\ t,\hfill &{{{{{{{\rm{Floquet}}}}}}}}\, - ({{{{{{{\rm{b}}}}}}}}) \hfill\\ {L}^{d},\hfill &{{{{{{{\rm{TI}}}}}}}}\, - ({{{{{{{\rm{c}}}}}}}}) \hfill\\ t{L}^{d},&{{{{{{{\rm{TI}}}}}}}}\,+\,{{{{{{{\rm{Floq}}}}}}}}. - ({{{{{{{\rm{d}}}}}}}}) \hfill\end{array}\right.} .$$

The first two lines are standard and result from replacing the time evolution with a random matrix drawn from the circular unitary ensemble (CUE), either re-drawn at every time step (a), or repeated in time (b)^4^. The remaining lines of Eq. (2) can be understood observing that TI on a square lattice leads to *L*^*d*^ momentum sectors, modeled as independent unitary blocks (still drawn from the CUE). This justifies the factors of *L*^*d*^ for cases (c) and (d).

**Fig. 2:**
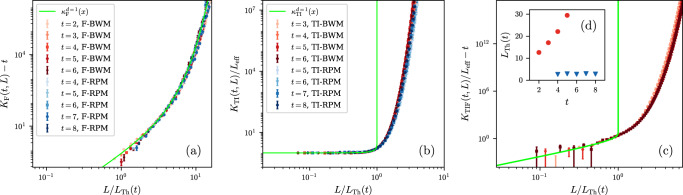
Numerical simulations of scaling forms of SFF for MBQC systems with space-time translational invariance. Scaled SFF vs. *x* = *L*/*L*_Th_ at finite *q* for **a** Floquet RPM and BWM, **b** TI RPM and BWM (middle), and **c** TI Floquet BWM; in all panels the RPM has *q* = 3, while the BWM has *q* = 2 in **a**, **b**, and *q* = 3 in **c**. The infinite-*q* scaling forms are plotted in green. **c** shows *t* = 2, 3, 4 from light to dark red. **d** *L*_Th_ is plotted against *t* for TIF BWM (red) and TIF RPM (blue). The TIF RPM is not shown in **c** because the apparent *L*_Th_(*t*) is too small (as seen in the inset) to be reliably estimated (see Supplementary Information (SI) for details).

Additionally, we characterize the corrections to RMT at large but finite *t* ≳ *t*_Th_(*L*). Our diagrammatic calculations at infinite-q indicate the existence of scaling forms in the scaling limit where both time *t* and the system size *L* are large but the ratio *L*/*L*_Th_(*t*) is kept fixed. Here, *L*_Th_(*t*) denotes the *Thouless length*, defined as the inverse function of the aforementioned Thouless time, i.e. *L*_Th_(*t*_Th_(*L*)) = *L*. Specifically, we obtain for the relevant cases (b), (c) and (d), the scaling forms

3 $$\begin{array}{l}\mathop{\lim }\limits_{\begin{array}{c}L,t\to \infty \\ L/{L}_{{{{{{{{\rm{Th}}}}}}}}}(t)=x\end{array}}{K}_{{{{{{{{\rm{F}}}}}}}}}(t,L)-t={\kappa }_{{{{{{{{\rm{F}}}}}}}}}(x),\\ \mathop{\lim }\limits_{\begin{array}{c}L,t\to \infty \\ L/{L}_{{{{{{{{\rm{Th}}}}}}}}}(t)=x\end{array}}{L}^{-1}{K}_{{{{{{{{\rm{TI}}}}}}}}}={\kappa }_{{{{{{{{\rm{TI}}}}}}}}}(x),\\ \mathop{\lim }\limits_{\begin{array}{c}L,t\to \infty \\ L/{L}_{{{{{{{{\rm{Th}}}}}}}}}(t)=x\end{array}}{L}^{-1}{K}_{{{{{{{{\rm{TIF}}}}}}}}}-t={\kappa }_{{{{{{{{\rm{TIF}}}}}}}}}(x).\end{array}$$

for *d* = 1, and for general *d* below in the "Methods” section. Remarkably, not only the validity of the scaling forms (3) obtained at *q* → *∞* is confirmed by our numerics at finite *q*, but we also have evidence that the value of the scaling functions *κ*(*x*) in each case is universal, being independent of the microscopic details of the model, which only affect the non-universal *L*_Th_(*t*). In Fig. 2, we numerically simulate (3) with two distinct random circuit models. While there are discrepencies between the finite-*q* data (red and blue) and infinite-*q* scaling function (light green) for TI and TIF cases, we see excellent scaling collapses for all cases, verifying (3).

## Discussion

The numerics shown in Fig. 2 (middle and right) shows a discrepancy between the infinite-*q* analytics, derived in "Methods” and SI, reproduced here,

4 $${\kappa }_{{{{{{{{\rm{F}}}}}}}}}^{d=1}(x)={e}^{x}-x-1,$$

5 $${\kappa }_{{{{{{{{\rm{TI}}}}}}}}}^{d=1}(x)={e}^{-x}(1-\ln (1-x)),$$

6 $${\kappa }_{{{{{{{{\rm{TIF}}}}}}}}}^{d=1}(x)=\ln \left(\frac{{e}^{-x}}{1-x}\right).$$

and the finite-*q* numerics in the presence of space translation invariance. There can be different justifications behind this discrepancy: One possibility is that finite *t* corrections decay very slowly for TI systems. This is qualitatively confirmed in the infinite-*q* case, inspecting how the limit in (5) is approached increasing *t* (see SI). A slow convergence is also to be expected due to the presence of singularities at finite *x* in ((5), (6)). More probably, we have indications that Eq. (4) is more robust than (5): By looking at the Floquet/TI RPM with *p*-site unit cell at infinite-q, we find that the scaling function (4) is independent of *p* while (5) is not (see SI). Still, it might appear puzzling that finite-*q* numerics shows a collapse to a scaling function indepedent of *q*, which is nonetheless not in agreement with the infinite-*q* analytics. To elucidate this aspect, we propose a simple qualitative scenario. First, we observe that in the RPM, the two parameters *ϵ* and *q* control respectively the coupling in the space and time directions and the RMT behavior emerges when long-range order is established in both directions. In *d* = 1, corrections to RMT are then controlled by dilute excitations which break ordered domains, either in space or time, i.e. in the leading order, we have *K*(*t*, *L*) ~ *K*_RMT_(*t*, *L*) + *g*_0_(*L*/*L*_Th,0_(*t*)) + *g*_1_(*L*/*L*_Th,1_(*t*)); where the subscript 0 and 1 refer respectively to the time and space directions, with the corresponding correlation lengths *L*_Th,0/1_(*t*). The functions *g*_0_(*x*) and *g*_1_(*x*) tend to zero as *x* → 0, and are expected to be model independent and only dependent on the symmetries. In the large *q* limit at fixed *L* and *t*, the coupling in the time direction becomes infinitely strong with ${L}_{{{{{{{{\rm{Th}}}}}}}},0}(t)\mathop{\longrightarrow }\limits^{q\to \infty }\infty$ so that only the function *g*_1_(*x*) survives in the decomposition above. For the Floquet RPM, we expect the relevant length scale to be *L*_Th_(*t*) ~ *L*_Th,1_(*t*) ≪ *L*_Th,0_(*t*), so that the scaling function is dominated by spatial domain walls, i.e. ${\kappa }_{{{{{{{{\rm{F}}}}}}}}}^{d=1}(x) \sim {g}_{1,{{{{{{{\rm{F}}}}}}}}}(x)$. However, for TI systems, *L*_Th_(*t*) ~ *L*_Th,0_(*t*) ≪ *L*_Th,1_(*t*), and therefore ${\kappa }_{{{{{{{{\rm{TI}}}}}}}}}^{d=1}(x) \sim {g}_{0,{{{{{{{\rm{TI}}}}}}}}}(x)$. However, as pointed out above, this scaling function is not accessible if *q* → *∞* before *L* and *t*, thus explaining the observed discrepancy between the numerics and the analytics. In practice, the universal scaling function *g*_0,TI_(*x*) observed in Fig. 2 results from *temporal domain walls* where contractions in different time slices take different permutation values. We stress that, because of unitarity, the SFF cannot diverge exponentially in *t*, therefore temporal domain walls must contribute *both* positively and negatively, in distinct contrast to the spatial domain walls discussed in^30,51^. This will be discussed in an upcoming work^53^.

A few additional comments are in order. Firstly, it would be beneficial to justify the universality which emerges from our work by means of a well-defined renormalization procedure. The main difficulty in this direction is the lack of locality and positivity of the resulting stat-mech model. Secondly, it is natural to expect that the scaling regime we identified is also visible in other quantities, as time-dependent correlation functions like out-of-time-ordered correlators. Thirdly, quasimomentum is conserved in TI lattice systems and affects its spectral properties but does not lead to the transport of an extensive conserved quantity because of Umklapp scattering; it is therefore interesting to explore its interplay with *U*(1) conserved charges. This will be discussed in an upcoming work^53^.

## Methods

### Models

The random phase model (RPM) is defined by a quantum circuit which is a matrix product $W(t)=\mathop{\prod }\nolimits_{t^{\prime}=1}^{t}w(t^{\prime} )$where *w*(*t*) = *w*_2_(*t*) *w*_1_(*t*) is a ${q}^{{{{{{{{\mathcal{N}}}}}}}}}\times {q}^{{{{{{{{\mathcal{N}}}}}}}}}$ operator, with ${{{{{{{\mathcal{N}}}}}}}}$ the number of sites. The model can be defined on arbitrary lattices, but here we focus on integer lattices ${{{{{{{\mathcal{L}}}}}}}}$ in *d*-dimension with periodic boundary conditions and length *L*, so that ${{{{{{{\mathcal{L}}}}}}}}\equiv {{\mathbb{Z}}}_{L}^{d}$ and ${{{{{{{\mathcal{N}}}}}}}}={L}^{d}$. ${w}_{1}(t){=\bigotimes }_{{{{{{{{\bf{r}}}}}}}}\in {{{{{{{\mathcal{L}}}}}}}}}u({{{{{{{\bf{r}}}}}}}},t)$ generates transformations at each site, with *q* × *q* unitary matrices *u*(**r**, *t*) chosen from the CUE; *w*_2_(*t*) couples neighboring sites and is diagonal in the basis of site orbitals with matrix elements ${[{w}_{2}(t)]}_{{a}_{1},\ldots {a}_{{{{{{{{\mathcal{N}}}}}}}}};{a}_{1},\ldots {a}_{{{{{{{{\mathcal{N}}}}}}}}}}=\exp \left[\imath {\sum }_{\langle {{{{{{{\bf{r}}}}}}}},{{{{{{{\bf{r}}}}}}}}^{\prime} \rangle }{\varphi }_{{a}_{{{{{{{{\bf{r}}}}}}}}}{,a}_{{{{{{{{\bf{r}}}}}}}}^{\prime} }}({{{{{{{\bf{r}}}}}}}},{{{{{{{\bf{r}}}}}}}}^{\prime},t)\right]$, where $\langle {{{{{{{\bf{r}}}}}}}},{{{{{{{\bf{r}}}}}}}}^{\prime} \rangle$ are the nearest neighbors in ${{{{{{{\mathcal{L}}}}}}}}$ and *a*_**r**_ ∈ {1, …, *q*}. We take each coefficient ${\varphi }_{{a}_{{{{{{{{\bf{r}}}}}}}}},{a}_{{{{{{{{\bf{r}}}}}}}}^{\prime} }}({{{{{{{\bf{r}}}}}}}},{{{{{{{\bf{r}}}}}}}}^{\prime},t)$ to be a Gaussian random variable with mean zero and variance *ϵ*, which effectively controls the coupling between neighboring spins.

For the temporally and spatially random RPM (Fig. 1a), all unitaries *u*(**r**, *t*) and phases $\varphi ({{{{{{{\bf{r}}}}}}}},{{{{{{{\bf{r}}}}}}}}^{\prime},t)$ are drawn independently. Correlation exists only between unitaries in ${{{{{{{\rm{Tr}}}}}}}}[W(t)]$ and their *unique* conjugates in ${{{{{{{\rm{Tr}}}}}}}}[{W}^{{{{\dagger}}} }(t)]$, which gives *K*(*t*, *L*) = 1 for all *q*. In^30^, the Floquet RPM (Fig. 1b) was considered where all gates are drawn independently in space but are constant in *t*. Here, we also consider the TI RPM (Fig. 1c), where the gates are such that $u({{{{{{{\bf{r}}}}}}}},t)=u(t),\phi ({{{{{{{\bf{r}}}}}}}},{{{{{{{\bf{r}}}}}}}}^{\prime},t)={\phi }^{(\mu )}(t)$, whenever ${{{{{{{\bf{r}}}}}}}}-{{{{{{{\bf{r}}}}}}}}^{\prime}={{{{{{{{\bf{e}}}}}}}}}_{\mu }$, with **e**_*μ*_ the unit vector in the *μ* spatial direction. The Floquet TI RPM (Fig. 1d), where the gates are also constant in time, is arguably the most realistic set-up.

### Numerics

To test the universality of the scaling forms in (3), we numerically evaluate the SFF for RPM and additionally the *brick wall model* (BWM) defined in the SI at finite *q* = 2, 3, *t* ≤ 8, and large *L*. Because of the computational cost, higher *d* are currently out-of reach and we restrict to *d* = 1. The comparison between two different models is shown in Fig. 2, together with the infinite-*q* analytical predictions shown in green (see ((10), (13), (16)) below). In all cases, we see an excellent collapse among the different models and times, consistent with the scaling form and pointing at the existence of a universal scaling function. We stress that the only free parameter in this procedure is the Thouless length *L*_Th_(*t*), which rescales the horizontal axis for each *t*. In our procedure, we fix it by imposing that the numerical data at different *t*’s all cross at a reference value ${x}_{0}=\tilde{L}/{L}_{{{{{{{{\rm{Th}}}}}}}}}(t)$ and equal the infinite-*q* expression (see SI). For Floquet circuits (Fig. 2a), using (3), we obtain an exceptionally good collapse for both models with the analytic infinite-*q* calculation (see (13) below). Note that while the SFF for the Floquet RPM had already been computed in ref. 30, the universality of the corresponding scaling function in the scaling limit had not been observed before. However, for both TI (non-Floquet) and TI Floquet circuits (Fig. 2b, c), the scaling functions which emerge from numerical data are not well described by those computed at infinite-q. The physical mechanism behind the discrepancies are described in the “Discussion” above.

### Analytics

**Fig. 3:**
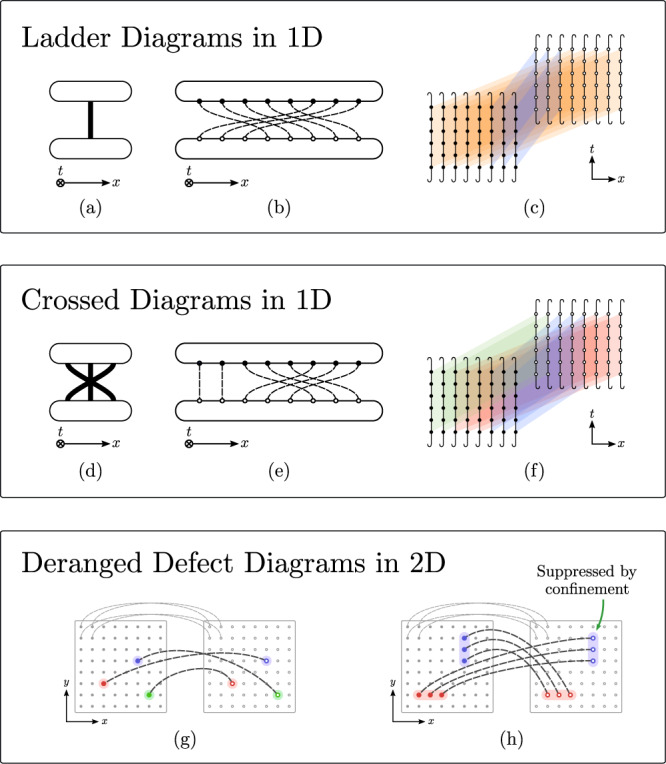
Many-body Feynman diagrams of SFF of MBQC systems with space-time translational invariance. **a** A representation of the set of ladder diagrams that appear in SFF of a TI RUC according to the rules presented in^29,30^. The top and bottom layers denote ${{{{{{{\rm{Tr}}}}}}}}[W(t)]$ and ${{{{{{{\rm{Tr}}}}}}}}[{W}^{{{{\dagger}}} }(t)]$ respectively. The thick line denote a group of parallel contractions between unitary gates and their conjugates (represented as dashed lines and dots in (**b**)). This set contains *t* ladder diagrams which are related to each other by a rigid translations of (say) the top layer, and are of the same order in *q*^29,30^. **b** A ladder diagram belonging to the set in (**a**). **c** A many-body Feynman diagram that contributes to the SFF of a TI RUC. The (colored) planes represent a group of parallel contractions. **d** The simplest set of crossed diagrams that appear in SFF for TI RUC in 1D. **e** A diagram belonging the set represented in **d**. **f** A many-body diagram that contributes to the SFF of TI RUC. **g** A spatial *deranged defect diagrams* that contributes to the SFF for TI RUC in 2D. The left and right square represents ${{{{{{{\rm{Tr}}}}}}}}[W(t)]$ and ${{{{{{{\rm{Tr}}}}}}}}[{W}^{{{{\dagger}}} }(t)]$ respectively. Most sites in ${{{{{{{\rm{Tr}}}}}}}}[W(t)]$ (dots in the left square) are contracted (gray dashed lines) with the same sites in ${{{{{{{\rm{Tr}}}}}}}}[{W}^{{{{\dagger}}} }(t)]$ (dots in the right square). We omit these contractions except four of them on the top left corner. The sites that do not contract with their counterpart are called *spatial defects* (labeled with colors). **h** Another crossed diagrams that appear in SFF for TI RUC in 2D. The phenomenon of confinement suppresses this diagram with a factor of *e*^−*ϵt*∂^, where ∂ is the size of the boundary of the defects (colored in red and blue).

Now we sketch the exact large-*q* analytics of SFF for the TI, Floquet, and TI FLoquet cases systematically. The analytics allows us to derive the emergence of the RMT behavior (2) in large-*t*, and the existence of scaling forms (3) describing the approach towards such emergence. Remarkably, as demonstrated in the numerics, the scaling forms are largely universal and depend only on *q*, spatial dimensionality, and the space-time symmetries. The dependence of universality classes in the spatial dimensionality was first observed in^30^ for Floquet systems and is even more striking for TI ones: In *d* = 1, corrections are controlled by *crossed diagrams* where sub-intervals in the top layer are rigidly contracted with those in the bottom layer (Fig. 3 d–f); instead, in *d*≥2, corrections are generated by *deranged defect diagrams*, where confinement forces excitations (on top of ladder diagrams) to be dilute (Fig. 3 g, h).

#### Translational invariant case

Here we compute the SFF of the TI–RPM in the limit *q* → *∞*. To compute the SFF, we perform the ensemble averages in two steps: (i) ensemble average over the CUE-s $u({{{{{{{\bf{r}}}}}}}},t^{\prime} )$; (ii) ensemble averages over the random phases. Within a fixed time slice $t^{\prime}$, there are ${{{{{{{\mathcal{N}}}}}}}}$ copies of $u({{{{{{{\bf{r}}}}}}}},t^{\prime} )$ / ${u}^{{{{\dagger}}} }({{{{{{{\bf{r}}}}}}}}^{\prime},t^{\prime} )$ on the top/bottom layer. Following^29^ (see SI), at the leading order in large *q* (throughout the manuscript, we will always take the order of limits where the limit of large *q* is taken before the limit of large *t* and *L* are taken), the ensemble average is expressed as a sum over permutations, $\sigma \in S({{{{{{{\mathcal{N}}}}}}}})$, pairing $u({{{{{{{\bf{r}}}}}}}},t^{\prime} )$ with ${u}^{{{{\dagger}}} }(\sigma ({{{{{{{\bf{r}}}}}}}}),t^{\prime} )$. Additionally, at leading order in large *q*, one is forced to take the same permutation on all *t* time slices, i.e. SFF is a single sum over $\sigma \in S({{{{{{{\mathcal{N}}}}}}}})$ (see SI). We now turn to the average over the random phases and we will see that it is natural to interpret it as a *cost function* associated to each *σ*. Expanding the orbital sum from phases at time slice $t^{\prime}$, we have ${\sum }_{{a}_{1},\ldots,{a}_{{{{{{{{\mathcal{N}}}}}}}}}}\exp \left[\imath {\sum }_{\langle {{{{{{{\bf{r}}}}}}}},{{{{{{{\bf{r}}}}}}}}^{\prime} \rangle }{\varphi }_{{a}_{{{{{{{{\bf{r}}}}}}}}},{a}_{{{{{{{{\bf{r}}}}}}}}^{\prime} }}(t^{\prime} )-{\varphi }_{{a}_{\sigma ({{{{{{{\bf{r}}}}}}}})},{a}_{\sigma ({{{{{{{\bf{r}}}}}}}})}}(t^{\prime} )\right]$ where the sum is over nearest neighbor pair of sites. We see that, in large *q*, cancellations of the phases are only possible whenever *σ* maps nearest neighbor sites onto nearest neighbors (preserving the orientation). Using that 〈*e*^ı*φ*^〉 = *e*^−*ϵ*/2^ and that all time slices contribute equally, we arrive at the expression

7 $${K}_{{{{{{{{\rm{TI}}}}}}}}}=\mathop{\sum}\limits_{\sigma \in {{{{{{{{\mathcal{S}}}}}}}}}_{{{{{{{{\mathcal{N}}}}}}}}}}{e}^{-\epsilon t(d{{{{{{{\mathcal{N}}}}}}}}-{N}_{{{{{{{{\rm{pb}}}}}}}}}(\sigma ))}$$

where *N*_pb_(*σ*) = *#*{**r**, *μ* ∣ *σ*(**r** + **e**_*μ*_) − *σ*(**r**) = **e**_*μ*_} is the number of *preserved bonds* in any direction by the permutation *σ* (*#* denotes the cardinality of a set). The sum in Eq. (7) can be reorganized by grouping all $\sigma \in {S}_{{{{{{{{\mathcal{N}}}}}}}}}$ with the same *N*_pb_(*σ*). We observe that *N*_pb_(*σ*) = *N*_pb_(*τσ*) for all translations $\tau \in {S}_{{{{{{{{\mathcal{N}}}}}}}}}$. Since the subgroup of translations is isomorphic to the lattice itself ${{{{{{{\mathcal{L}}}}}}}}$, we arrive at

8 $${K}_{{{{{{{{\rm{TI}}}}}}}}}={{{{{{{\mathcal{N}}}}}}}}\mathop{\sum }\limits_{n=0}^{d{{{{{{{\mathcal{N}}}}}}}}}{A}_{d}({{{{{{{\mathcal{N}}}}}}}},n){e}^{-n\epsilon t},$$

where ${A}_{d}({{{{{{{\mathcal{N}}}}}}}}, n)=\#\{\sigma \in {S}_{{{{{{{{\mathcal{N}}}}}}}}}/{{{{{{{\mathcal{L}}}}}}}}\,|\,{N}_{{{{{{{{\rm{pb}}}}}}}}}(\sigma )=d{{{{{{{\mathcal{N}}}}}}}}-n\}$ and $n=d{{{{{{{\mathcal{N}}}}}}}}-{N}_{{{{{{{{\rm{pb}}}}}}}}}(\sigma )$ is the number of *broken bonds*. Computing exactly the ${A}_{d}({{{{{{{\mathcal{N}}}}}}}},n)$ poses a non-trivial combinatorial problem, which nevertheless simplifies in *d* = 1 or for large ${{{{{{{\mathcal{N}}}}}}}}$, as we show below. However, it is easy to see that ${A}_{d}({{{{{{{\mathcal{N}}}}}}}},n=0)=1$, corresponding to the identity equivalence class. Therefore in the limit *t* → *∞*, we recover the RMT result for this case in Eq. (2). Generalizing this construction, one can see that *K* converges to the dimension of the group of spatial symmetries. As example, for a two-dimensional TI circuit on a square lattice with rotational symmetry by angles of *π*/2, ${K}_{{{{{{{{\rm{TI}}}}}}}}}={{{{{{{\mathcal{N}}}}}}}}=4$ in the limit of large *t*.

For *d* = 1, $\sigma \in {S}_{L}/{{\mathbb{Z}}}_{L}$ can be represented as cyclic permutations of *L* elements: Represent $\sigma \in {S}_{L}/{{\mathbb{Z}}}_{L}$ as $\sigma ^{\prime} \equiv (\sigma (1),\sigma (2),\ldots )$ and define an associated cyclic permutation with cycle $\sigma ^{\prime}$. Then, *σ* with a fixed number of broken bonds *n* can be obtained as follows: We first partition ${{{{{{{\mathcal{L}}}}}}}}\equiv {{\mathbb{Z}}}_{L}={I}_{1}\cup {I}_{2}\ldots {I}_{n}$ into *n* adjacent non-empty intervals. Then we take any cyclic permutation $\tilde{\sigma }$ of *n* elements such that $\tilde{\sigma }(i) \, \ne \, \tilde{\sigma }(i+1)+1\,({{{{{{\mathrm{mod}}}}}}}\,\,n)$ and rigidly map ${I}_{i}\to {I}_{\tilde{\sigma }(i)}$. Clearly, the resulting mapping breaks exactly *n* bonds and all possible mappings can be uniquely constructed in this way. As an example, see Fig. 3e where *L* = 8, *n* = 3 and $\tilde{\sigma }=(132)$. This leads to

9 $${A}_{1}(L,n)=\left({{L}\atop{n}}\right) {a}_{n}$$

where the binomial factor counts the partitionings of ${{{{{{{\mathcal{L}}}}}}}}$ and *a*_*n*_ the possible $\tilde{\sigma }$. Although an explicit expression for the *a*_*n*_ is not available, they correspond to a well-studied sequence whose exponential generating function is known^54,55^. For both *L*, *t* large with fixed *x* = *L*/*L*_Th_(*t*) < 1, *L*_Th_(*t*) = *e*^*ϵt*^, we can take $\left({{L}\atop{n}}\right) {a}_{n} \sim {L}^{n}/n!$ using the dominated convergence theorem^56^ and sum over *n* to obtain the scaling form

10 $$\mathop{\lim }\limits_{\begin{array}{c}L,t\to \infty \\ L/{L}_{{{{{{{{\rm{Th}}}}}}}}}(t)=x\end{array}}{L}^{-1}{K}_{{{{{{{{\rm{TI}}}}}}}}}^{d=1}={e}^{-x}(1-\ln (1-x))\equiv {\kappa }_{{{{{{{{\rm{TI}}}}}}}}}^{d=1}(x).$$

*L*_Th_(*t*) denotes the Thouless length. Note also that throughout the article, the limit of large *q* is always taken before the limits of large *t* and *L*.

For *d* > 1, evaluating the multiplicities ${A}_{d}({{{{{{{\mathcal{N}}}}}}}},n)$ is a much harder task as they result from the interplay between permutations and the geometry of ${{{{{{{\mathcal{L}}}}}}}}$. Nevertheless, the problem simplifies in the limit of large *L* at fixed *n*, as it corresponds to a dilute regime where a fixed number of bonds is broken in a very large system. Consider first a transposition which exchanges two sites. This will generally break 4*d* bonds (2*d* neighbors for each site), and therefore ${A}_{d \,{ > }\,1}({{{{{\mathcal{N}}}}}},4d) \sim {{{{{{\mathcal{N}}}}}}}^{2}/2$. More generally, we first pick the positions of *k* well-separated spatial defects and then we consider the possible ways of permuting them without leaving fixed points, so that precisely *n* = 2*dk* bonds are broken (Fig. 3g). These *deranged defect diagrams* lead to the asymptotic expansion

11 $${A}_{d}({{{{{{{\mathcal{N}}}}}}}},n=2dk)\mathop{=}\limits^{{{{{{{{\mathcal{N}}}}}}}}\to \infty }\frac{{{{{{{{{\mathcal{N}}}}}}}}}^{k}}{k!}{d}_{k}\,,\quad d \, > \, 1$$

where *d*_*k*_ are the number of *derangements*^55,57^ (i.e. permutation with no fixed points) of *k* elements. The error we make in (11) is related to situations where the defects are close to one another thus breaking less bonds, but these are sub-leading in ${{{{{{{\mathcal{N}}}}}}}}$. Once again, we consider the limit *t*, *L* → *∞* at fixed $x={{{{{{{\mathcal{N}}}}}}}}/{{{{{{{{\mathcal{N}}}}}}}}}_{{{{{{{{\rm{Th}}}}}}}}}(t)$, with ${{{{{{{{\mathcal{N}}}}}}}}}_{{{{{{{{\rm{Th}}}}}}}}}(t)={e}^{2d\epsilon t}$ the *Thouless volume*, and obtain

12 $$\mathop{\lim }\limits_{\begin{array}{c}L,t\to \infty \\ {{{{{{{\mathcal{N}}}}}}}}/{{{{{{{{\mathcal{N}}}}}}}}}_{{{{{{{{\rm{Th}}}}}}}}}(t)=x\end{array}}{{{{{{{{\mathcal{N}}}}}}}}}^{-1}{K}_{{{{{{{{\rm{TI}}}}}}}}}^{d \,{ > }\,1}=\frac{{e}^{-x}}{1-x}\equiv {\kappa }_{{{{{{{{\rm{TI}}}}}}}}}^{d \,{ > }\,1}(x)$$

We stress that the apparent difference between the scaling functions (10) and (12) has a fundamental origin: In *d* = 1, extended intervals can be rigidly exchanged paying a cost only at their boundary; instead, in *d* > 1, exchanging two extended domains has a cost which grows with their boundary, i.e. extended defects are suppressed by *confinement*. Therefore, the leading contribution at large *L* and *t* is given by well-separated single-site excitations.

#### Floquet case

Before analyzing the effect of combining time-periodicity and translation invariance, we review the calculation of *K*_F_(*t*, *L*) for the Floquet RPM (Fig. 1a)^30^. Here the single-site unitary gates are constant in time but are random in space *u*(**r**, *t*) = *u*(**r**). Thus, in the diagrammatic expansion of the SFF, we can choose any permutation *σ* ∈ *S*_*t*_ to pair the *t*–copies of *u*(**r**) in the top layer with those of *u*^†^(**r**) in the bottom one. In the limit of large *q*, only time translations contribute, i.e. ${\sigma }_{{\mathsf{t}}}(k)=k+{\mathsf{t}}\,({{{{{{\mathrm{mod}}}}}}}\,\,t)$ with *t* = 0, …, *t* − 1^30^. Therefore, we get a many-body diagram by choosing a configuration *t*(**r**) (color) for each site ${{{{{{{\bf{r}}}}}}}}\in {{{{{{{\mathcal{L}}}}}}}}$. After averaging over the random phases, it was shown that *K*_F_(*t*, *L*) = *Z*_Potts_, with *Z*_Potts_ the partition function of a *t*-state Potts model with a Boltzmann weight across all bonds ${{{{{{{{\mathcal{W}}}}}}}}}_{{\mathsf{t}},{\mathsf{t}}^{\prime} }={e}^{-\epsilon t(1-{\delta }_{{\mathsf{t}},{\mathsf{t}}^{\prime} })}$. At large *t*, the partition function is dominated by the *t* ferromagnetic groundstates where all sites have the same color, leading to the RMT prediction *K*_F_ ~ *t*. As *t* approaches *t*_Th_ from above, excitations from the *t* ferromagnetic groundstates – the lowest-lying excitation being the domain wall states^30^ – become important. To access such corrections, in 1D, one makes use of the transfer matrix to write ${Z}_{{{{{{{{\rm{Potts}}}}}}}}}={{{{{{{\rm{Tr}}}}}}}}[{{{{{{{{\mathcal{W}}}}}}}}}^{L}]$. Computing the spectrum of ${{{{{{{\mathcal{W}}}}}}}}$ and evaluating *Z*_Potts_ in the scaling limit, this leads to the scaling form (see SI)

13 $$\mathop{\lim }\limits_{\begin{array}{c}L,t\to \infty \\ L/{L}_{{{{{{{{\rm{Th}}}}}}}}}(t)=x\end{array}}{K}_{{{{{{{{\rm{F}}}}}}}}}^{d=1}-t={e}^{x}-x-1\equiv {\kappa }_{{{{{{{{\rm{F}}}}}}}}}^{d=1}(x)$$

with *x* = *L*/*L*_Th_(*t*) and *L*_Th_(*t*) = *e*^*ϵt*^/*t*. In higher dimension, *Z*_Potts_ cannot be easily computed for finite *L* and *t*. Nevertheless, in the scaling limit where *L*, *t* are both large, corrections to the RMT SFF are associated with diluted excitations where the color is changed with respect to the ground state’s one. The position of the excitation can be chosen in $\sim {{{{{{{{\mathcal{N}}}}}}}}}^{n}/n!$ ways and each of them can be assigned any of the *t* − 1 remaining color, with a cost *e*^−2*nϵdt*^. As a consequence, setting $x={{{{{{{\mathcal{N}}}}}}}}/{{{{{{{{\mathcal{N}}}}}}}}}_{{{{{{{{\rm{Th}}}}}}}}}(t)$ and ${{{{{{{{\mathcal{N}}}}}}}}}_{{{{{{{{\rm{Th}}}}}}}}}(t)={e}^{2d\epsilon t}/t$,

14 $$\mathop{\lim }\limits_{\begin{array}{c}L,t\to \infty \\ {{{{{{{\mathcal{N}}}}}}}}/{{{{{{{{\mathcal{N}}}}}}}}}_{{{{{{{{\rm{Th}}}}}}}}}(t)=x\end{array}}{t}^{-1}{K}_{{{{{{{{\rm{F}}}}}}}}}^{d \,{ > }\,1}={e}^{x}\equiv {\kappa }_{{{{{{{{\rm{F}}}}}}}}}^{d \,{ > }\,1}(x).$$

Intriguingly, note that, in contrast with Eqs. (10) and (12) which are divergent for any *x* ≥ 1, Eqs. (13) and (14) remain always smooth for finite *x*. This can be understood observing that infinite-*q* TI systems are mapped onto stat-mech models with non-local interactions, so that the scaling function is associated with the exchange of distant domains (*d* = 1) or defects (*d* > 1); on the contrary for Floquet systems, the resulting Potts model has purely local interactions.

#### Translational invariant Floquet case

We can now turn to the TI Floquet case. The same matrix CUE matrix *u* and *u*^†^ appear $t{{{{{{{\mathcal{N}}}}}}}}$ times respectively in the top and bottom layer. However, at large *q* only the subgroup ${S}_{{{{{{{{\mathcal{N}}}}}}}}}\times {{\mathbb{Z}}}_{t}^{{{{{{{{\mathcal{N}}}}}}}}}\subset {S}_{t{{{{{{{\mathcal{N}}}}}}}}}$, corresponding to arbitrary spatial permutations *σ* and time translations *t*(**r**) at each site. In *d* = 1, as explained in the TI case, the permutation *σ* corresponds to crossed diagrams, where spatial intervals in the top layer ${{{{{{{\rm{Tr}}}}}}}}[W(t)]$ are mapped onto intervals in the bottom one ${{{{{{{\rm{Tr}}}}}}}}[{W}^{{{{\dagger}}} }(t)]$ (e.g. Fig. 3e). Then, the cost associated to the average over the phases depends on the choices of *t*(**r**): within the same interval, the cost is given by the Boltzmann weights ${{{{{{{\mathcal{W}}}}}}}}$ as in the Floquet case; instead, between different intervals, the cost is always *e*^−*ϵt*^ irrespectively of the choice of *t*’s at the interface. To account for the resulting combinatorics, it is useful to introduce the partition function $Z(\omega )={{{{{{{\rm{Tr}}}}}}}}({({{{{{{{\mathcal{W}}}}}}}}+\omega {{{{{{{\mathcal{R}}}}}}}})}^{L})={\sum }_{n}{\omega }^{n}{Z}_{n}(t,L)$, where ${{{{{{{{\mathcal{R}}}}}}}}}_{{\mathsf{t}},{\mathsf{t}}^{\prime} }={e}^{-\epsilon t}$ is a rank 1 matrix with constant coefficients. In words, *n* counts the number of intervals and the factors *Z*_*n*_(*t*, *L*) contain the sum over all possible the colors with a *n* intervals. This leads to the explicit formula valid for arbirtrary *t* and *L*

15 $${K}_{{{{{{{{\rm{TIF}}}}}}}}}^{d=1}(t,L)=\mathop{\sum }\limits_{n=0}^{\infty }{a}_{n}{Z}_{n}(t,L).$$

In the scaling limit, we obtain the behavior (see SI)

16 $$\mathop{\lim }\limits_{\begin{array}{c}L,t\to \infty \\ L/{L}_{{{{{{{{\rm{Th}}}}}}}}}(t)=x\end{array}}{L}^{-1}{K}_{{{{{{{{\rm{TIF}}}}}}}}}^{d=1}-t=\ln \left(\frac{{e}^{-x}}{1-x}\right)\equiv {\kappa }_{{{{{{{{\rm{TIF}}}}}}}}}^{d=1}(x).$$

In *d* > 1, the large-*t* dominant contribution corresponds to ladder diagrams in space and a ferromagnetic ground state in the color, leading as expected to ${K}_{{{{{{{{\rm{TIF}}}}}}}}}^{d \,{ > }\,1}(t,L) \sim t{L}^{d}$. Corrections at large *L*, *t* are once again obtained by diluted excitations which can have two different origins: derangements as in (12), which are now *deranged defect diagrams* in space-time; or color changes as in (14). The two effects combine multiplicatively (see SI)

17 $$\mathop{\lim }\limits_{\begin{array}{c}L,t\to \infty \\ {{{{{{{\mathcal{N}}}}}}}}/{{{{{{{{\mathcal{N}}}}}}}}}_{{{{{{{{\rm{Th}}}}}}}}}(t)=x\end{array}}{({{{{{{{\mathcal{N}}}}}}}}t)}^{-1}{K}_{{{{{{{{\rm{TIF}}}}}}}}}^{d \,{ > }\,1}=\frac{1}{1-x}\equiv {\kappa }_{{{{{{{{\rm{TIF}}}}}}}}}^{d \,{ > }\,1}(x),$$

with $${{{{{{{{\mathcal{N}}}}}}}}}_{{{{{{{{\rm{Th}}}}}}}}}(t)={e}^{2d\epsilon t}/t$$.

## Supplementary information

Supplementary information

## Data Availability

All relevant data are available from the authors.

## Code availability

Codes used for this study are available upon request by contacting the authors.
