# Supplementary material for: Many-body quantum chaos and space-time translational invariance
Source: Nat Commun. 2022 Dec 5;13:7484. doi: 10.1038/s41467-022-34318-1 (PMC9722696; doi:10.1038/s41467-022-34318-1)
Supplement: Supplementary file 1 — Supplementary information [file 41467_2022_34318_MOESM1_ESM.pdf]

Supplementary Information

Many-Body Quantum Chaos and Space-time Translational Invariance

Amos Chan, Saumya Shivam, David A. Huse, and Andrea De Luca

In this supplementary information we provide additional details about:

A) SFF for translational invariant RPM

1. Derivation of Eq. 7
2. One dimensional case
3. Higher dimensional case

B) SFF for Floquet RPM.

1. One dimensional case
2. Higher dimensional case

C) SFF for translational invariant Floquet RPM

1. One dimensional case
2. Higher dimensional case

D) Generalised unit cell in large-q

1. SFF for TI RPM with p-site translational invariance
2. SFF for Floquet RPM with p-discrete-time translational invariance

E) Brick wall model (BWM)

F) Numerical methods and results

1. SFF for translational invariant circuits
2. SFF for Floquet circuits
3. SFF for translational invariant Floquet circuits
4. Comparison between scaling forms and finite-t, finite-L, and infinite-q solutions

Appendix A: SFF for translational invariant RPM

1. Derivation of Eq. 7

To derive Eq. 7, we compute the ensemble average of the TI-RPM in two steps: the ensemble average of (i) the unitaries drawn from the CUE in $w_1(t')$, and (ii) the random phases in $w_2(t')$. To carry out (i), we recall that the ensemble averages of unitaries $u_{i,j}$ drawn the CUE can be evaluated as $\langle u_{i_1 j_1} u_{i_2 j_2} \dots u_{i_\ell j_\ell} u^*_{i'_1,j'_1} u^*_{i'_2,j'_2} \dots u^*_{i'_\ell,j'_\ell} \rangle = \sum_{\sigma,\tau\in S_\ell} \mathrm{Wg}(\sigma^{-1}\tau, q) \prod_{k=1}^{\ell} \delta_{i_{\sigma(k)},i'_k} \delta_{j_{\tau(k)},j'_k}$ where S_ℓ is the symmetric group of ℓ elements, and $\mathrm{Wg}(\sigma^{-1}\tau, q)$ is the Weingarten function [60, 61]. $\mathrm{Wg}(\sigma^{-1}\tau, q)$ can be expressed as a polynomial in $1/q$, and is of the leading order in q when $\sigma = \tau$. We apply the above formula to average over unitaries in a fixed time slice t'. In the large-q limit, the sum over permutation is dominated by the term with $\sigma = \tau \in S_{\mathcal{N}}$, where $\mathcal{N}$ is the number of sites in the lattice. Together with all t time slices, the ensemble average is a sum over a vector of permutations, $\vec{\sigma} = (\sigma_1, \sigma_2, \dots, \sigma_t)$, where $\sigma_i \in S_{\mathcal{N}}$. Observe that the average over the random phases can only maintain or decrease the order in $1/q$ of a given contribution. This implies that in the large-q limit, the leading contribution must have $\sigma_1 = \sigma_2 = \cdots = \sigma_t = \sigma \in S_{\mathcal{N}}$, so that the number of sums over the site orbitals is maximized. Next, we perform the ensemble averages of the random phases in (ii). Expanding the orbital sum from phases at time slice t', we have

$$\sum_{\{\mathbf{a}\},\{\mathbf{a}'\}=1}^{q} \prod_{\alpha,\beta=1}^{q} \int D\varphi_{\alpha\beta} \prod_{\mathbf{r}}^{\mathcal{N}} e^{\imath \sum_{\mu} \left[\varphi_{a_{\mathbf{r}} a_{\mathbf{r}+\mathbf{e}_\mu}} - \varphi_{a'_{\mathbf{r}} a'_{\mathbf{r}+\mathbf{e}_\mu}} \right]} \delta_{a_{\mathbf{r}}, a'_{\sigma(\mathbf{r})}} , \tag{SA.1}$$

where $D\varphi_{\alpha\beta} = \left[\exp\left(-\frac{\varphi_{\alpha\beta}^2}{2\epsilon}\right)/\sqrt{2\pi\epsilon}\right] d\varphi_{\alpha\beta}$, and where $\{\mathbf{a}\}$ and $\{\mathbf{a}'\}$ labels the orbital degrees of freedom in $\mathrm{Tr}[W(t)]$ and $\mathrm{Tr}[W^\dagger(t)]$ respectively. The delta functions in (SA.1) will lead to cancellations of phases in the exponent, when $\sigma(\mathbf{r}+\mathbf{e}_\mu) - \sigma(\mathbf{r}) = \mathbf{e}_\mu$. In the large-$q$ limit, each remaining phase $\varphi_{a_\mathbf{r} a_{\mathbf{r}+\mathbf{e}_\mu}}$ will give rise to a factor of $\langle e^{\imath\varphi}\rangle = e^{-\epsilon/2}$. Furthermore, since all time slices take the same contraction σ, the factors from (SA.1) are raised to the t-th power. This gives us the expression Eq. (7).

2. One dimensional case

Consider first of all a possible partitioning of $\mathcal{L}$ into n intervals $I_1, \ldots, I_n$. We define the set

$$M_n = \{\tilde{\sigma} \in S_n/\mathbb{Z}_n | \forall i\ , \quad \tilde{\sigma}(i+1) \neq \sigma(i) + 1 \pmod{n}\}\ . \tag{SA.2}$$

As explained in the main text, we denote as a_n its cardinality: $a_n = \# M_n$. The sequence a_n satisfies

$$\sum_n \frac{a_n z^n}{n!} = e^{-z}(1 - \ln(1-z))\ , \tag{SA.3}$$

which converges for all $|z| < 1$. Therefore, taking L large in Eq. (9), we have

$$\lim_{\substack{L,t\to\infty \\ L/L_{\mathrm{Th}}(t)=x}} L^{-1} K_{\mathrm{TI}}^{d=1} = \sum_{n=0}^{\infty} \frac{a_n x^n}{n!} = e^{-x}(1-\ln(1-x)) \equiv \kappa_{\mathrm{TI}}^{d=1}(x)\ , \tag{SA.4}$$

where $x = L/L_{\mathrm{Th}}(t)$ and $L_{\mathrm{Th}}(t) = e^{\epsilon t}$, as given in Eq. (10) of the main text.

$x \geq 1$ *regime*

According to the analysis in the previous section, we saw that the scaling function $\kappa_{\mathrm{TI}}^{d=1}(x)$ diverges when $x \geq 1$. We can investigate how does it diverge at finite but large L. In order to do so, we consider the exact expression for finite L at infinite q and $d = 1$, in Eqs. (7) and (9).

$$\kappa_{\mathrm{TI}}^{d=1}(x; L) \equiv \sum_{n=0}^{L} \binom{L}{n} a_n x^n L^{-n}\ , \tag{SA.5}$$

where we fixed $x = L/L_{\mathrm{Th}}$. Since for $x \geq 1$, the expression in (SA.5) diverges at large L, its behavior is dominated by the large n expansion of the a_n coefficients which can be deduced from (SA.3) and reads

$$a_n \simeq \frac{n!}{e(n+1)} \equiv \tilde{a}_n \tag{SA.6}$$

We can thus split the sum (SA.5) as

$$\kappa_{\mathrm{TI}}^{d=1}(x; L) \simeq \sum_{n=0}^{L_0} \binom{L}{n} (a_n - \tilde{a}_n) x^n L^{-n} + \sum_{n=0}^{L} \binom{L}{n} \tilde{a}_n x^n L^{-n}\ , \tag{SA.7}$$

where L_0 is large but finite. The first term in (SA.7) converges to the finite contribution

$$\sum_{n=0}^{L_0} \binom{L}{n} (a_n - \tilde{a}_n) x^n L^{-n} \quad \overset{L\to\infty}{\longrightarrow} \quad \sum_{n=0}^{L_0} \frac{(a_n - \tilde{a}_n) x^n}{n!} \tag{SA.8}$$

To evaluate the second term in (SA.7), we use the integral representation of the factorial as

$$n! = \int_0^\infty dt\, e^{-t} t^n \tag{SA.9}$$

which leads to

$$\sum_{n=0}^{L}\binom{L}{n}\tilde{a}_n x^n L^{-n} = \frac{L}{ex(L+1)}\int_0^\infty \frac{dt}{t}\, e^{-Lt}\left((1+tx)^{L+1}-1\right) \tag{SA.10}$$

For large L and $x > 1$, this last integral can be estimated via saddle point at $t^* = (x-1)/x$, which leads to

$$\kappa_{\mathrm{TI}}^{d=1}(x; L) \propto \exp[L(\ln x - 1 + 1/x)] \tag{SA.11}$$

For $x = 1$, the integral is dominated by small $t^* \propto 1/L$, which leads eventually to

$$\kappa_{\mathrm{TI}}^{d=1}(x; L) \simeq \frac{\ln L}{2e} + O(1) \tag{SA.12}$$

3. Higher dimensional case

For higher dimension, we cannot obtain an explicit expression for $K_{\mathrm{TI}}^{d>1}$ at finite $\mathcal{N}$ and t. However, we are still interested in the limit of large L and t. In this case, once again we are interested in the dilute limit, where a permutation only exchanges a fixed number of sites without leaving fixed points. For the number of derangements d_n, we have the exponential generating function

$$\sum_{n=0}^{\infty}\frac{d_n z^n}{n!} = \frac{e^{-x}}{1-x}\ . \tag{SA.13}$$

Then, using (8) and (11) in the main text, we arrive at

$$\lim_{\substack{L,t\to\infty \\ \mathcal{N}/\mathcal{N}_{\mathrm{Th}}(t)=x}} \mathcal{N}^{-1} K_{\mathrm{TI}}^{d>1}(t, L) = \sum_{n=0}^{\infty}\frac{x^n d_n}{n!} = \frac{e^{-x}}{1-x} \equiv \kappa_{\mathrm{TI}}^{d>1}(x)\ , \tag{SA.14}$$

where $x = \mathcal{N}/\mathcal{N}_{\mathrm{Th}}(t)$ and $\mathcal{N}_{\mathrm{Th}}(t) = e^{2d\epsilon t}$, as given in Eq. (12) of the main text.

Appendix B: SFF for the Floquet RPM

As reviewed in the main text, the calculation of the SFF in the Floquet RPM [30] at $q \to \infty$ can always be mapped into the partition function of a corresponding Potts model

$$K_{\mathrm{F}}(t, L) = Z_{\mathrm{Potts}}\ , \tag{SB.1}$$

whose Boltzmann weights are given by the matrix $W_{\mathsf{t},\mathsf{t}'} = e^{-\epsilon t(1-\delta_{\mathsf{t},\mathsf{t}'})}$ and $\mathsf{t}, \mathsf{t}' = 0, \ldots, t-1$.

1. One dimensional case

In 1d, we can simply express the partition function by using the Transfer matrix

$$K_{\mathrm{F}}^{d=1}(t, L) = \mathrm{Tr}[\mathcal{W}^L]\ . \tag{SB.2}$$

The spectrum of the matrix $\mathcal{W}$ contains two eigenvalues $\lambda_+ > \lambda_-$: $\lambda_- = 1 - \exp(-\epsilon t)$ with degeneracy $t-1$ and $\lambda_+ = 1 + e^{-t\epsilon}(t-1)$. Consequently,

$$K_{\mathrm{F}}^{d=1}(t, L) = (t-1)\lambda_-(t)^L + \lambda_+(t)^L\ . \tag{SB.3}$$

In this case, setting $L_{\mathrm{Th}}(t) = e^{\epsilon t}/t$ and $x = L/L_{\mathrm{Th}}(t)$, we have

$$\lim_{\substack{L,t\to\infty \\ L/L_{\mathrm{Th}}(t)=x}} K_{\mathrm{F}}^{d=1}(t, L) - t = e^x - x - 1 \equiv \kappa_{\mathrm{F}}^{d=1}(x)\ , \tag{SB.4}$$

as given in Eq. (13) of the main text.

2. Higher dimensional case

For $d > 1$, the calculation of the Potts partition function poses a non-trivial problem. For $d = 2$, integrability can be used. However, here we are interested in the scaling limit where both the number of sites $\mathcal{N}$ and the time t are large. Let's set $z = e^{-\epsilon t}$ and consider the small z (large ϵ) expansion. We focus on $d = 2$ for simplicity, but the procedure can be easily extended to any $d > 1$. This corresponds to a low-temperature expansion in the ferromagnetic phase. At the zero-th order, Z_{Potts} is simply given by the t possible groundstates. The leading correction is obtained changing the color of one site. This can be done on any site, choosing any among the $t-1$ remaining colors and will break 4 bonds; the corresponding contribution to the partition function is therefore $\mathcal{N}(t-1)z^4$. Higher orders in z are obtaining changing colors at more sites. For instance, considering two site changes, we have two cases, according to whether the two sites are nearest neighbours or not. The first case gives a contribution $4(t-1)\mathcal{N}z^6 + 4(t-1)(t-2)\mathcal{N}z^7$. The second case gives instead $\mathcal{N}(\mathcal{N}-5)(t-1)^2z^8$. In the limit, $L, t \to \infty$ with fixed $x = \mathcal{N}tz^4 = \mathcal{N}/\mathcal{N}_{\text{Th}}(t)$, we clearly see that only the last contribution survives. This corresponds to a dilute limit, which once accounting for the permutations among defects, takes in any $d > 1$ the form

$$\lim_{\substack{L,t\to\infty \\ \mathcal{N}/\mathcal{N}_{\text{Th}}(t)=x}} t^{-1}K_{\text{F}}^{d>1}(t,L) = \sum_{n=0}^{\infty} \frac{x^n}{n!} = e^x \equiv \kappa_{\text{F}}^{d>1}(x) \;, \tag{SB.5}$$

with $x = \mathcal{N}/\mathcal{N}_{\text{Th}}$ $\mathcal{N}_{\text{Th}}(t) = z^{-2d}/t = e^{2d\epsilon t}/t$, as given in Eq. (14) of the main text.

Appendix C: SFF for translational invariant Floquet RPM

In the TI Floquet RPM, the diagrams appearing in the expansion of the SFF can be put in correspondence of i) spatial permutations σ; ii) time translations at each site. We can thus face the problem in two steps, first fixing the permutation σ and then summing over the possible choices of time translations at each site with fixed σ. We analyse separately the 1d from the $d > 1$ case.

1. One dimensional case

As explained for the temporal random case, fixing the permutation σ is equivalent to partitioning the L sites into intervals $I_1, \ldots, I_n$ and then mapping the intervals on the top layer onto intervals on the bottom layer using cyclic permutations $\tilde{\sigma} \in M_n$ in (SA.2). For a fixed choice of the interval and of $\tilde{\sigma}$, the sum over the possible choices of the time translations can be still written in terms of a Potts-like partition function with modified Boltzmann weights: bonds inside the same interval are given by the matrix $\mathcal{W}$; bonds at the interface between two different intervals always give the trivial Boltzmann weight $e^{-\epsilon t}$. Since choosing a partition $I_1, \ldots, I_n$ can be done choosing the bonds where the edges of the intervals are, we can rewrite the SFF introducing the generalised partition function

$$Z(\omega) = \text{Tr}[(\mathcal{W} + \omega\mathcal{R})^L] = \sum_{n=0}^{\infty} \omega^n Z_n \;, \tag{SC.1}$$

where we introduced the rank 1 matrix $\mathcal{R}_{\text{t,t}'} = e^{-\epsilon t}$. The coefficients Z_n in the power series expansion of $Z(\omega)$ contain all the configurations where n intervals are present and thus the trivial Boltzmann weight $\mathcal{R}_{\text{t,t}'}$ is used. We thus have

$$K_{\text{TIF}}^{d=1}(t,L) = L\sum_{n=0}^{L} a_n Z_n \;, \tag{SC.2}$$

where, as before, a_n is defined as the cardinality of the set in (SA.2). The coefficients Z_n can be computed explicitly from the spectrum of $\mathcal{W} + \omega\mathcal{R}$. Similarly to (SB.3), we have

$$Z(\omega) = (t-1)\lambda_-(t,\omega)^L + \lambda_+(t,\omega)^L \tag{SC.3}$$

where $\lambda_-(t,\omega) = \lambda_-(t) = 1 - e^{-\epsilon t}$ and $\lambda_+(t,\omega) = 1 + e^{-t\epsilon}((t-1) + \omega t)$. It follows that

$$Z_0 = K_{\text{F}}^{d=1}(t,L) \;, \qquad Z_n = \lambda_+(t,\omega=0)^L \binom{L}{n} \left(\frac{t}{e^{t\epsilon}+t-1}\right)^n \;, \quad n > 1 \;. \tag{SC.4}$$

Taking once again the scaling limit, we obtain

$$\lim_{\substack{L,t\to\infty \\ L/L_{\rm Th}(t)=x}} L^{-1}K^{d=1}_{\rm TIF}(t,L) - t = \kappa^{d=1}_{\rm F}(x) + e^{x}(\kappa^{d=1}_{\rm TI}(x)-1) = -x - \ln(1-x) \equiv \kappa^{d=1}_{\rm TIF}(x) \tag{SC.5}$$

with $x = L/L_{\rm Th}(t)$ and $L_{\rm Th}(t) = e^{t\epsilon}/t$, as given in Eq. (16) of the main text.

2. Higher dimensional case

An explicit formula for the SFF at finite t and L is hard to derive in this case, because it would require the computation of the Potts model partition function in d-dimensions where some bonds have been removed. We focus on the scaling limit $L, t \to \infty$ but with $x = \mathcal{N}/\mathcal{N}_{\rm Th}(t)$ kept constant. As explained above this correspond to a dilute limit. We thus have the expansion

$$K^{d>1}_{\rm TIF}(t,L) \sim t\mathcal{N}\left[\sum_{n=0}^{\infty}\frac{(t\mathcal{N})^n}{n!}d_n e^{-2d\epsilon nt}\right]\left[\sum_{m=0}^{\infty}\frac{\mathcal{N}^m}{m!}(t-1)^n e^{-2d\epsilon mt}\right]. \tag{SC.6}$$

The origins of the expression are explained as follows: In the first square bracket, the sum over n accounts for the deranged defects on top of the rigid spatial translation which can be chosen in $\binom{N}{n} \sim N^n/n!$ ways. The factor of t accounts for t choices of the $\mathtt{t}$ variables for these defects, and the factor d_n counts their derangements. In the second square bracket, the sum over m accounts for the defects in the sum over the $\mathtt{t}$ variables. We can choose m defects in $\binom{\mathcal{N}}{m} \sim \mathcal{N}^m/m!$ ways and each of them can be independently changed with the $t-1$ other colours different from the background. Taking again the scaling limit

$$\lim_{\substack{L,t\to\infty \\ \mathcal{N}/\mathcal{N}_{\rm th}(t)=x}} (t\mathcal{N})^{-1}K^{d>1}_{\rm TIF}(t,L) = \kappa^{d>1}_{\rm F}(x)\times\kappa^{d>1}_{\rm TI}(x) = \frac{1}{1-x} \equiv \kappa^{d>1}_{\rm TIF}(x)\,, \tag{SC.7}$$

with $\mathcal{N}_{\rm Th}(t) = e^{2d\epsilon t}/t$ and $x = \mathcal{N}/\mathcal{N}_{\rm Th}(t)$, as given in Eq. (17) of the main text.

Appendix D: Generalised unit cell in large-q

In this appendix, we demonstrate the robustness of the scaling forms. To this end, we compute the scaling forms in the large-q limit of the modified circuit models, namely TI RPM (Floquet RPM) that are invariant under p-site (p-discrete-time) translation in space (time). The 2-site and 2-discrete-time translational invariant model is illustrated in Fig. S1a and b respectively. A summary of the results is as follows. For TI RPM with p-site translational invariance, we show that for $d=1$, the scaling form $\kappa^{(p)}_{\rm TI}$ is lower bounded by $\kappa^{(1)}_{\rm TI}$ in Eq. (SD.6). For $d\geq 2$, we show that $\kappa^{(p)}_{\rm TI} = \left[\kappa^{(1)}_{\rm TI}(x)\right]^p$ in Eq. (SD.9). For Floquet RPM with p-discrete-time translational invariance, we show that for all dimension d that $\kappa^{(p)}_{\rm F}(x) = \kappa^{(1)}_{\rm F}(x)$ in Eq. (SD.11) and (SD.13).

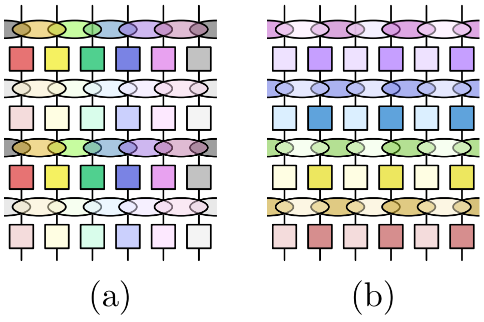

FIG. S1. **Illustrations of RUC with larger translational invariant unit cell.** Illustrations of (a) Floquet (and spatially random) RPM which is invariant under 2-discrete-time translation; and (b) TI (and temporally random) RPM which is invariant under 2-site translation. For each case, gates of the same colors are identical.

1. SFF for TI RPM with p-site translational invariance

One-dimensional systems

For $d = 1$, we derive a lower bound for $K_{\rm TI}^{(p)}$ (and $\kappa_{\rm TI}^{(p)}$) for TI RPM with p-site translational invariance. We will first present the derivation for $p = 2$ then generalise it to any $p \in \mathbb{N}^+$. Following the derivation of Eq. (8), we have for $p = 2$, a sum over two permutations, σ_1 and σ_2

$$K_{\rm TI}^{(2)}(t, L_{\rm eff}) = \sum_{\substack{\sigma_1 \in \mathcal{S}_{\rm odd} \\ \sigma_2 \in \mathcal{S}_{\rm even}}} e^{-\epsilon t (L - N_{\rm pb}(\tilde{\sigma}[\sigma_1, \sigma_2]))} \ , \tag{SD.1}$$

where $L_{\rm eff} = L/2$ is the number of unit cells, $\mathcal{S}_{\rm odd}$ / $\mathcal{S}_{\rm even}$ are permutations restricted to the odd/even sublattices and $N_{\rm pb}(\sigma)$ is the number of preserved bonds as defined in the main text. The permutation $\tilde{\sigma}$ is defined composing σ_1 and σ_2

$$\tilde{\sigma}[\sigma_1, \sigma_2](i) = \begin{cases} \sigma_1(i) & \text{if } i \text{ is odd} \\ \sigma_2(i) & \text{if } i \text{ is even} \end{cases} \tag{SD.2}$$

It is difficult to evaluate (SD.1) exactly, but a lower bound can be identified. Observe that one can always restrict the broken bonds to be placed in between site i and $i+1$ for even i. This means $K_{\rm TI}^{(2)} > L_{\rm eff} \sum_{n=0}^{L_{\rm eff}} A_1(L_{\rm eff}, n)$, where A_1 is given in Eq. (9) in the main text. In addition to the above configurations, one can find another set of diagrams where the broken bonds are placed between site i and $i+1$ with odd i. Putting the two cases together and accounting for the overcounting of the cases where no bonds are broken we have

$$K_{\rm TI}^{(2)}(t, L_{\rm eff})/L_{\rm eff} \geq \sum_{n_1=0}^{L_{\rm eff}} \binom{L_{\rm eff}}{n_1} a_{n_1} e^{-n_1 \epsilon t} + \sum_{n_2=0}^{L_{\rm eff}} \binom{L_{\rm eff}}{n_2} a_{n_2} e^{-n_2 \epsilon t} - 1 \tag{SD.3}$$

This expression is easily generalised to arbitrary p to

$$K_{\rm TI}^{(p)}(t, L_{\rm eff})/L_{\rm eff} \geq p \sum_{n=0}^{L_{\rm eff}} \binom{L_{\rm eff}}{n} a_n e^{-n \epsilon t} - (p-1) \tag{SD.4}$$

with $L_{\rm eff} = L/p$ in general.

In the scaling limit where $t, L \to \infty$ with fixed $x = L_{\rm eff}/L_{\rm Th} = L_{\rm eff} e^{-\epsilon t}$, we define

$$\kappa_{\rm TI}^{(p)}(x) \equiv \lim_{\substack{L, t \to \infty \\ L_{\rm eff}/L_{\rm Th}(t) = x}} L_{\rm eff}^{-1} K_{\rm TI}^{(p)}(t, L) \ . \tag{SD.5}$$

Note that with this definition $\kappa_{\rm TI}^{(p)}(x = 0) = 1$, which corresponds to the RMT behavior at large times. From (SD.4), we obtain the lower bound for the scaling function

$$\kappa_{\rm TI}^{(p)}(x) \geq p \kappa_{\rm TI}^{(1)}(x) - (p-1) \ , \tag{SD.6}$$

Note that this equation implies the existence of a divergence at $x = 1$ for all p's.

Higher-dimensional systems

For $d > 1$, with the foresight that we will use the scaling limit where only dilute deranged defects matter, we define a generic higher-dimensional TI RPM as follows: Consider a TI RPM made up of super-sites of p qudits, each of which evolve under independently drawn CUE, and couple with z other qudits under the (independently drawn) random phase gates defined in the main text. Additionally, we demand that the translational invariance of the supersites is preserved.

Consider the scaling limit where $t, L \to \infty$ at fixed $x = \mathcal{N}_{\rm eff}/\mathcal{N}_{\rm Th}$ with $\mathcal{N}_{\rm eff} = \mathcal{N}/p$ and $\mathcal{N}_{\rm Th} = e^{z\epsilon t}$. In this limit, SFF is a sum over deranged defect diagrams labelled by $(k_1, k_2, \ldots, k_p)$ where k_i is the number of deranged dilute

defects of the i-th type of qudits in the supersite. Following the derivation of Eq. (8), (11) and (12), in the limit of large $\mathcal{N}$, we write

$$\mathcal{N}_{\text{eff}}^{-1} K_{\text{TI}}^{(p)} \sim \prod_{i=1}^{p} \sum_{k_i=0}^{\mathcal{N}_{\text{eff}}} \frac{d_{k_i} \mathcal{N}_{\text{eff}}^{k_i}}{k_i! \mathcal{N}_{\text{Th}}^{k_i}} = \left[\mathcal{N}_{\text{eff}}^{-1} K_{\text{TI}}^{(1)}\right]^p , \tag{SD.7}$$

where again d_k is the number of derangements of k elements. Using the definitions

$$\kappa_{\text{TI}}^{(p)}(x) \equiv \lim_{\substack{\mathcal{N},t\to\infty \\ \mathcal{N}_{\text{eff}}/\mathcal{N}_{\text{Th}}(t)=x}} \mathcal{N}_{\text{eff}}^{-1} K_{\text{TI}}^{(p)}(t,L) , \tag{SD.8}$$

we arrive a more compact statement in the scaling limit

$$\kappa_{\text{TI}}^{(p)} = \left[\kappa_{\text{TI}}^{(1)}(x)\right]^p . \tag{SD.9}$$

Again, with this convention we have $\kappa_{\text{TI}}^{(p)}(x=0) = 1$, which corresponds to the RMT behavior at large times.

2. SFF for Floquet RPM with p-discrete-time translational invariance

The calculation of SFF for RPM with p-discrete-time translation invariance closely follows the one for RPM with 1-discrete-time translation invariance (see main text and [29, 30]). For all dimensions d, upon averaging over the CUE in the large-q limit following [29, 30], SFF is mapped to a Potts model with DOF t_i at each site i. t_i can take $t_{\text{eff}} = t/p$ number of possible states, corresponding to the t_{eff} possible ladder diagrams. The averages over the random phases give an effective Boltzmann weight $\mathcal{W}_{\mathsf{t},\mathsf{t}'} = e^{-t_{\text{eff}}(1-\delta_{\mathsf{t},\mathsf{t}'})\epsilon_{\text{eff}}}$ with $\epsilon_{\text{eff}} = \epsilon/p$ for nearest neighbour pairs of state t and t'. Therefore, the SFF $K_{\text{F}}^{(p)}(t_{\text{eff}}, L)$ can be written as the partition function of a t_{eff}-state Potts model with Boltzmann weight parametrized by ϵ_{eff}.

One-dimensional systems

In $d = 1$, we define in the scaling limit,

$$\kappa_{\text{F}}^{(p)}(x) \equiv \lim_{\substack{L,t\to\infty \\ \mathcal{N}/\mathcal{N}_{\text{Th}}(t)=x}} K_{\text{F}}^{(p)}(t_{\text{eff}}, L) - t_{\text{eff}} . \tag{SD.10}$$

where $L_{\text{Th}}(t_{\text{eff}}) = e^{t_{\text{eff}}\epsilon_{\text{eff}}}/t_{\text{eff}} = pe^{t\epsilon}/t$. $\kappa_{\text{F}}^{(p)}(x)$ can now be related to $\kappa_{\text{F}}^{(1)}(x)$ as

$$\kappa_{\text{F}}^{(p)}(x) = e^x - x - 1 = \kappa_{\text{F}}^{(1)}(x) . \tag{SD.11}$$

In other words, after properly defining the Thouless length, the scaling function is the same independently of p. Note that at large time, we have $\kappa_{\text{F}}(x=0) = 0$, and the RMT result is reproduced.

Higher-dimensional systems

For $d \geq 1$, we define $\mathcal{N}_{\text{Th}} = e^{z\epsilon_{\text{eff}}t_{\text{eff}}}/t_{\text{eff}}$ with $t_{\text{eff}} = t/p$ and $\epsilon_{\text{eff}} = p\epsilon$. Furthermore, we define

$$\kappa_{\text{F}}^{(p)}(x) \equiv \lim_{\substack{L,t\to\infty \\ \mathcal{N}/\mathcal{N}_{\text{Th}}(t)=x}} t_{\text{eff}}^{-1} K_{\text{F}}^{(p)}(t_{\text{eff}}, L) . \tag{SD.12}$$

In the scaling limit, $\kappa_{\text{F}}^{(p)}$ coincides with $\kappa_{\text{F}}^{(1)}$ as

$$\kappa_{\text{F}}^{(p)}(x) = \kappa_{\text{F}}^{(1)}(x) , \tag{SD.13}$$

i.e. the scaling function is independent of p. Note again that at large time, we have $\kappa_{\text{F}}(x=0) = 0$, and the RMT result is reproduced.

Appendix E: Brick wall model (BWM)

The one-dimensional BWM is defined by a quantum circuit which is a matrix product

$$W(t) = \prod_{t'=1}^{t} w(t') \tag{SE.1}$$

where $w(t) = w_2(t)\, w_1(t)$ is a $q^L \times q^L$ operator.

$$w_1(t) = \bigotimes_{n=1}^{L/2} u_{2n-1,2n}(t) \tag{SE.2}$$

is a tensor product of $q^2 \times q^2$ unitary matrices $u_{2n-1,2n}(t)$ chosen from the circular unitary ensemble (CUE) and acting on site $2n-1$ and $2n$.

$$w_2(t) = \bigotimes_{n=1}^{L/2} u_{2n,2n+1}(t) \tag{SE.3}$$

is again a tensor product of $u_{2n,2n+1}(t)$ drawn from CUE except that the unitary gate acts on site $2n$ and $2n+1$. The model is defined with periodic boundary condition with $u_{L,L+1}(t)$ acting on site L and 1.

For the temporally and spatially random BWM, each unitary gate $u_{n,n+1}(t)$ is drawn independently. For Floquet BWM, we take unitary gates acting on different pairs of sites to be independently drawn, while gates acting on the same pair of sites at different discrete time t to be identically drawn, i.e. $w(t) = w(t')$ for $t \neq t'$ and $W(t) = w^t$. For TI BWM, gates in $w_1(t)$ (and separately in $w_2(t)$) acting on different pairs of sites at the same time are identically drawn, but gates acting on different discrete times are independently drawn. For Floquet TI BWM, $u_{n,n+1}(t) = u_{n',n'+1}(t')$ for even integer n and for all t. The equation separately holds true for odd integer n.

Appendix F: Numerical methods and results

We simulate the RPM with $q = 3$, $\epsilon = 2$ and $d = 1$, and the BWM with $q = 2, 3$, both in $d = 1$ and with periodic boundary conditions. (We find that RPM with $q = 2$ and large ϵ is not fully chaotic in the sense that the linear ramp does not appear in late t.) We consider two types of numerical simulations: "Time-direction" simulations and "space- (or dual-)direction" simulations, which involve performing calculations by acting matrices in the Hilbert space $\mathcal{H} = \bigotimes^L \mathbb{C}^q$ and the dual Hilbert space $\tilde{\mathcal{H}} = \bigotimes^t \mathbb{C}^q$ respectively. Although Figure 2 uses only dual direction numerics, here we discuss both the directions.

For the time-direction simulations with translational invariant (but not Floquet) circuits, the computation of $K(t, L)$ vs t for different L involves sparse matrix multiplication of locally-supported random unitaries, and taking the trace. For translational invariant Floquet circuits, we compute $K(t, L)$ for a fixed L with exact diagonalizations (ED).

For the space-direction simulations, we define a dual Floquet operator V, which we explicitly construct for RPM. V for BWM can be similar constructed following the procedure below. Informally, V is taken to be the tensor product of the first column of tensors in Fig. 1b and 1c. More precisely, we introduce the computational basis $\mathbf{b} = \{b^1, \ldots, b^t\}$ with each $b^\mu = 1, \ldots, q$. The dual tensor for RPM can now be written as

$$[v_1]_{\mathbf{b},\mathbf{b}'} = \prod_{\mu=1}^{t} e^{\imath \varphi_{b^\mu, b^{\mu\prime}}} \,, \tag{SF.1a}$$

$$[v_2]_{\mathbf{b},\mathbf{b}'} = \prod_{\mu=1}^{t} u_{b^{\mu+1}, b^\mu} \delta_{\mathbf{b},\mathbf{b}'} \,, \tag{SF.1b}$$

so that $V = v_2 v_1$ and $V(L) = V^L$. Note that in the dual formulation the 1-body unitary matrices in w_1 are converted into 2-body diagonal matrices in v_2, while the 2-body phases in w_2 are converted into the 1-body v_1. Because of periodic boundary conditions and taking the trace, the SFF for the dual Floquet operator can also be expressed as a trace,

$$K(t, L) = \langle \mathrm{Tr}_{\mathcal{H}}[W(t)]\, \mathrm{Tr}_{\mathcal{H}}[W^\dagger(t)] \rangle = \langle \mathrm{Tr}_{\tilde{\mathcal{H}}}[V(L)]\, \mathrm{Tr}_{\tilde{\mathcal{H}}}[V^\dagger(L)] \rangle \,, \tag{SF.2}$$

where we have added subscripts to the traces to emphasize the Hilbert spaces on which matrices W and V act. The dual tensors for TI BWM, with a computational basis $\mathbf{b} = \{b^1, b^2 \ldots, b^{2t}\}$, can be similarly written as

$$[v_1]_{\mathbf{b},\mathbf{b}'} = \prod_{\mu=1}^{t} \tilde{u}_{b^{2\mu},b^{2\mu+1}}(\mu) \; , \tag{SF.3a}$$

$$[v_2]_{\mathbf{b},\mathbf{b}'} = \prod_{\mu=1}^{t} \tilde{u}_{b^{2\mu-1},b^{2\mu}}(\mu) \; , \tag{SF.3b}$$

where $\tilde{u}_{ij,kl} = u_{jl,ik}$, $u_{ij,kl}$ being a CUE random matrix acting on two sites, and $\tilde{u}$ is the dual of the unitary gate, which is non-unitary in general. The dual Floquet operator is given by $V = v_2 v_1$ and $V(L) = V^{L/2}$.

1. SFF for translational invariant circuits

We elaborate on the middle panel of Figure 2, and provide further evidence of consistency with the predicted scaling form in Eq. 10. For both RPM and BWM, $K_{\mathrm{TI}}(t, L)$ is computed by exact diagonalizing the dual Floquet operator V. In figure S2, we re-plot the data for RPM (left), BWM with $q = 2$ (middle), and also add the data for BWM with $q = 3$ in the right panel (whose importance is spelled out in the discussion on TIF circuits), for which only three system sizes were accessible. Averaging was done over $12000 - 15000$ realizations of V for RPM, $8000 - 15000$ realizations for BWM with $q = 2$, and over $4000 - 10000$ realizations for BWM with $q = 3$.

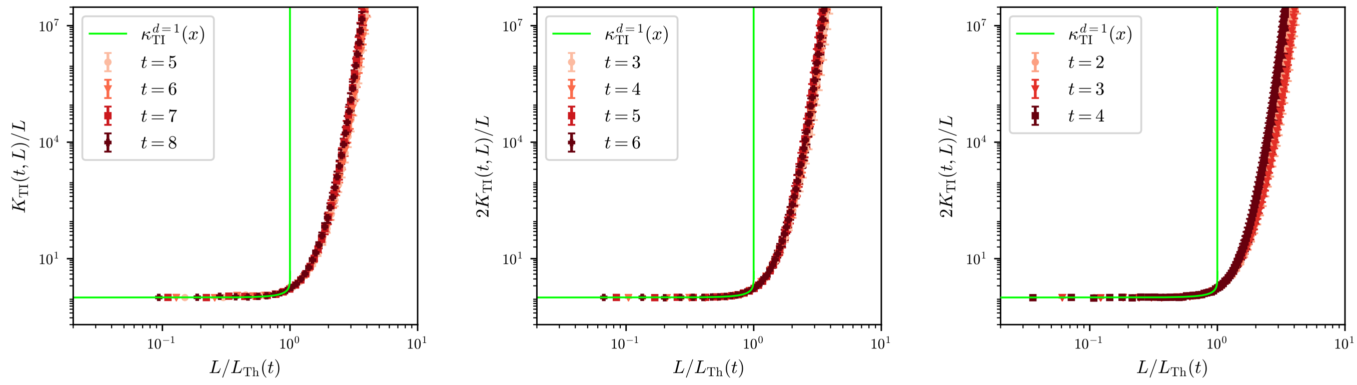

(a) (b) (c)

FIG. S2. **Additional numerics of the SFF scaling function of TI MBQC circuits.** $K(t, L)/L$ vs. L/L_{Th} for (a) TI-RPM at $q = 3$, (b) TI-BWM at $q = 2$, and (c) TI-BWM at $q = 3$.

Now we describe the details of how we obtain $L_{\mathrm{Th}}(t)$ and plot the collapse in Figure 2. The horizontal axes in 2 are all scaled such that the finite-q numerics for $K_{\mathrm{TI}}(t, L)/L$ is equal to the infinite-q scaling function $\kappa_{\mathrm{TI}}^{d=1}(x)$ (Eq. 10) at a specific point $x_0 < 1$. We choose $x_0 = 0.95$ and determine $\tilde{L}(t)$ for a given t such that $K_{\mathrm{TI}}(t, \tilde{L})/\tilde{L} = \kappa_{\mathrm{TI}}^{d=1}(x_0)$. Since $x_0 = \tilde{L}(t)/L_{\mathrm{Th}}(t)$ by definition, we get the corresponding Thouless time $L_{\mathrm{Th}}(t) = \tilde{L}(t)/x_0$. Lastly, we rescale the horizontal axis to be $x = L/L_{\mathrm{Th}}(t)$, so the numerics and the scaling function can be compared directly. We visually show this procedure in Figure S3, where the left panel shows the horizontal lines for a few x_0 that we draw to extract $\tilde{L}(t)$, and the right panel shows $L_{\mathrm{Th}}(t)$ vs t for those x_0. We find little difference between the behaviour at different x_0, and exponential fits to $L_{\mathrm{Th}}(t)$ are consistent with the expected scaling form in Eq. 10.

$L_{\mathrm{Th}}(t)$ for BWM (both with $q = 2$ and $q = 3$) were similarly calculated and is shown in the middle and the right panel of Figure S7.

As additional checks for consistency, we consider time direction simulations, using sparse matrix multiplication and trace evaluation, in Figure S4, where the left panel shows $K_{\mathrm{TI}}(t, L)/L$ vs t for different L, averaged over $6000 - 9000$ realizations of $W(t)$. Using this data, $t_{\mathrm{Th}}(L)$ can be obtained analogous to $L_{\mathrm{Th}}(t)$ by looking at the intersection of $K_{\mathrm{TI}}(t, L)/L$ with a constant $\delta = \kappa_{\mathrm{TI}}^{d=1}(x_0)$ for some x_0, as plotted in the middle panel. Note that here we are only

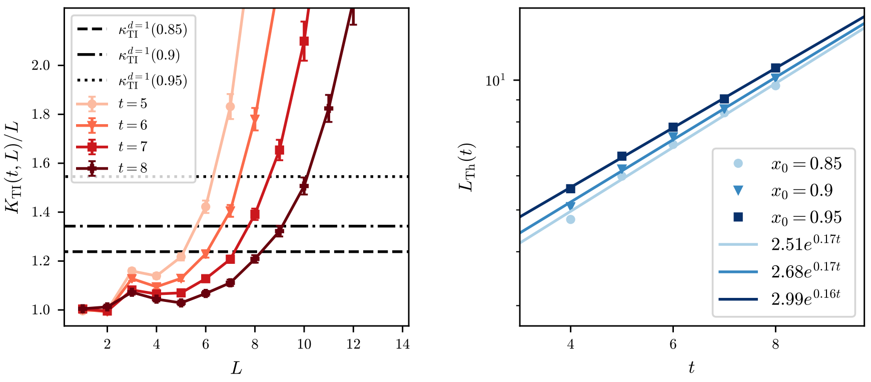

(a) (b)

FIG. S3. **Methods of obtaining $L_{\rm Th}(t)$ and the dependence of $L_{\rm Th}(t)$ on x_0.** (a): $K_{\rm TI}(t,L)/L$ vs L for different L using space direction simulations of TI RPM, with the intersections $\tilde{L}(t)$ with constant horizontal lines $\kappa_{\rm TI}^{d=1}(x_0)$ for a few x_0 used to compute $L_{\rm Th}(t) = \tilde{L}/x_0$. (b): $L_{\rm Th}(t)$ vs t for different choices of x_0. The corresponding exponential fits (solid lines) show reasonable agreement with the expected form from Eq. 10 ($L_{\rm Th} = e^{\epsilon_{\rm eff} t}$), with some effective $\epsilon_{\rm eff} \neq 2$ due to finite-q effects.

interested in the scaling form of $t_{\rm Th}(L)$ hence we ignore the normalization arising due to different δ. Good logarithmic fits indicate consistency with the form of $L_{\rm Th}(t) = Le^{-\epsilon_{\rm eff} t}$, where the fitted $\epsilon_{\rm eff} \neq 2$ is due to finite q effects. The scaling form is then further corroborated in the right panel, showing a collapse with $0.2t - \log(L)$, with $\epsilon_{\rm eff}$ being consistent across all plots.

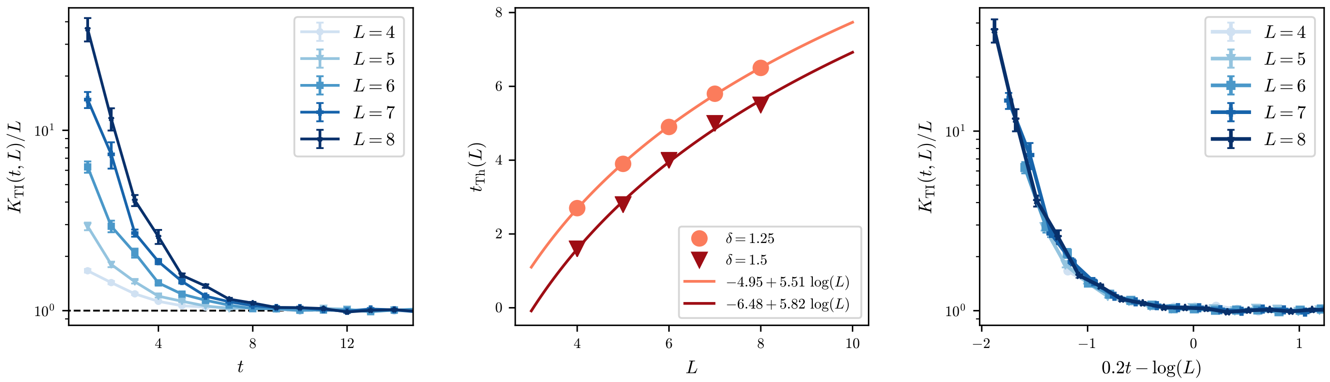

(a) (b) (c)

FIG. S4. **SFF of TI MBQC circuits.** (a): $K_{\rm TI}(t,L)/L$ vs t for different L obtained using time direction simulations of TI RPM. (b): $t_{\rm Th}(L)$ vs L, computed by numerically solving $K_{\rm TI}(t_{\rm Th}, L)/L = \delta$. A logarithmic fit is consistent with the expectation that $L_{\rm Th}(t) = Le^{-\epsilon_{\rm eff} t}$, with an effective $\epsilon_{\rm eff} \neq 2$ due to finite-q corrections, and also consistent with the fit in Fig. S3. (c): $K_{\rm TI}(t,L)/L$ vs $0.2t - \log(L)$.

2. SFF for Floquet circuits

Here, we elaborate on the left panel of Figure 2. We first show in Figure S5 the collapse of $K_{\rm F}(t,L) - t$ vs $L/L_{\rm Th}(t)$ separately for RPM with $q = 3, \epsilon = 2$ (left), BWM with $q = 2$ (middle), and additionally BWM with $q = 3$(right); and we find that for all these circuits, the infinite-q scaling function in Eq. 13 is in excellent agreement with finite-q numerics for these circuits. Note that these are all space direction simulations performed through sparse matrix

multiplication and trace computation and not through ED. The data for RPM has been averaged over $4000-10000$ realizations, and for BWM with $q=2$ over $3000-10000$ realizations, while for BWM with $q=3$, we averaged over $4000-10000$ realizations. $L_{\rm Th}(t)$ were obtained in a similar fashion as for TI RPM (Figure S3), fixing $x_0=3$, and are shown in the left panel of Figure S7.

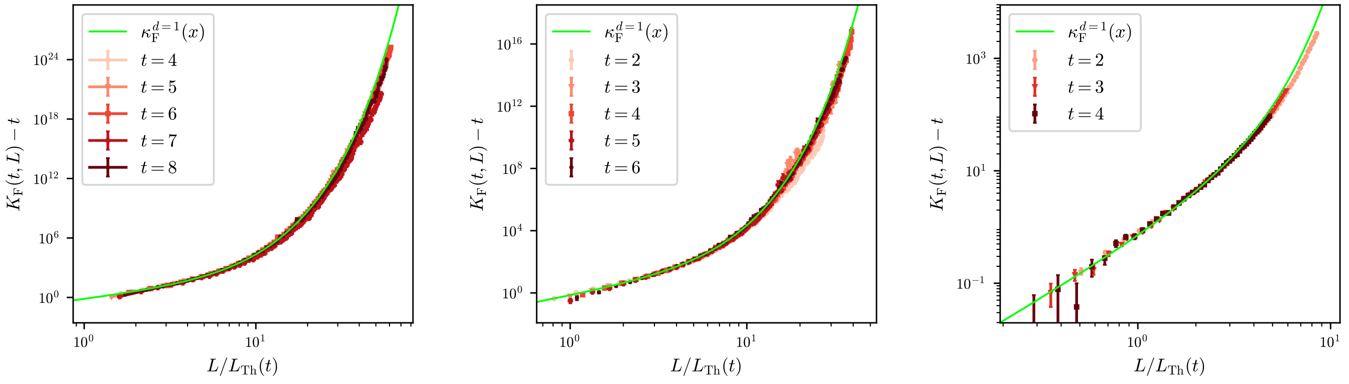

(a) (b) (c)

FIG. S5. **Additional numerics of the SFF scaling function of Floquet MBQC circuits.** $K(t,L)-t$ vs. $L/L_{\rm Th}$ for (a) F-RPM at $q=3$, (b) F-BWM at $q=2$, and (c) F-BWM at $q=3$.

3. SFF for translational invariant Floquet circuits

In this part of the Appendix, we focus on transnational invariant Floquet circuits, and elaborate on the right panel of Figure 2. First, we separately show in Figure S6, $K_{\rm TIF}/L-t$ vs $L/L_{\rm Th}(t)$ for RPM with $q=3,\epsilon=2$ (left), BWM with $q=2$ (middle), and BWM with $q=3$. The data is averaged over $8000-15000$ realizations of the dual Floquet operator V for RPM, $7000-1000$ realizations for BWM with $q=2$, and $4000-10000$ realizations for BWM with $q=3$. As can be seen in the figure, there aren't enough points for $x=L/L_{\rm Th}(t)<1$ for RPM with $q=3$ and BWM with $q=2$, in order to see a collapse for $x<1$, hence cannot be compared reliably for $x>1$ either. This can be seen as a consequence of small $L_{\rm Th}(t)$ for those two models (as shown in the right panel of Figure S7). TIF-RPM with $q=3,\epsilon=2$, in particular, has the additional problem where the extracted $L_{\rm Th}(t)$ in fact are probing the region beyond the RMT ramp, when $t>t_{\rm Hei}\approx q^L/L$. Note that this is not an issue for F-RPM, since $t_{\rm Hei}=q^L$ for that model. These problems are not present in the data for BWM with $q=3$ (right panel of Figure S7), because $L_{\rm Th}(t)$ are sufficiently large for the accessible values of t, allowing data points for $x<1$. Note that $L_{\rm Th}(t)$ for $t=5$ is obtained by stochastic sampling of the trace through multiplication with complex random vectors, and is not as accurate as the points for $t\leq 4$.

To further investigate on the dynamics of TIF-RPM with $q=3,\epsilon=2$, we look at $K_{\rm TIF}/L$ vs t using time direction simulations, and show that $t_{\rm Th}$ is of the order of $t_{\rm Hei}$ for accessible L. The left panel of Figure S8 plots $K_{\rm TIF}/L$ vs t, averaged over $8000-10000$ realizations of the Floquet operator W, along with the RMT behavior $K_{\rm RMT}$, given by

$$K_{\rm RMT}(t,L)=\begin{cases} tL & t<t_{\rm Hei}\\ q^L & t\geq t_{\rm Hei}\end{cases}. \tag{SF.4}$$

It can be observed in the middle panel that the $K_{\rm TIF}/L$ approaches $K_{\rm RMT}/L$ at times larger than $t_{\rm Hei}$. The middle panel shows a rolling average of the data from the plot in the left panel. To quantify $t_{\rm Th}$, one could draw a horizontal line near zero and look at the intersection of the difference between $K_{\rm TIF}/L$ and $K_{\rm RMT}/L$. A large $t_{\rm Th}$ is consistent with the small $L_{\rm Th}(t)$ we see in Figure S7, and is also reflected in the ratio of consecutive level spacings $r=\min(\delta_n,\delta_{n+1})/\max(\delta_n,\delta_{n+1})$, where $\delta_n=|\phi_n-\phi_{n-1}|$ is the difference between consecutive eigenphases $\{\phi_n\}$, which is plotted in the right panel, averaging over 100 realizations of the Floquet operator for each model. The plot shows that the spacing ratio for TIF-RPM hasn't converged to the GUE value for the accessible system sizes, unlike the other two models we have studied.

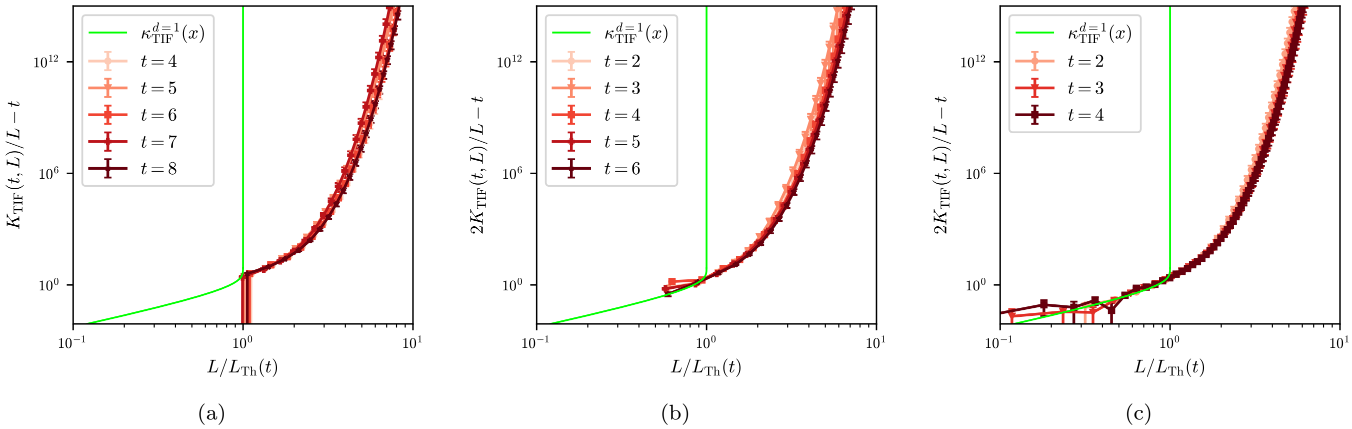

FIG. S6. **Additional numerics of the SFF scaling function of TI Floquet MBQC circuits.** $K(t,L)/L_{\text{eff}}-t$ vs. L/L_{Th} for (a) TIF-RPM at $q=3$, (b) TIF-BWM at $q=2$, and (c) TIF-BWM at $q=3$.

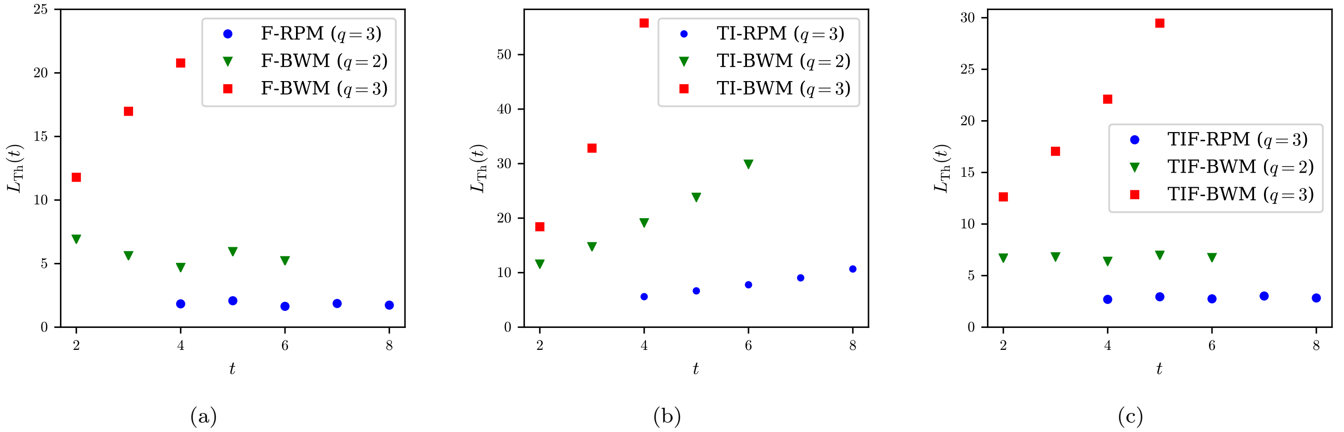

FIG. S7. **Dependence on L_{Th} on t for MBQC circuits with space-time translational invariance.** $L_{\text{Th}}(t)$ vs. t for (a) Floquet circuits, (b) TI circuits, and (c) TI Floquet circuits.

4. Comparison between scaling forms and finite-t, finite-L, and infinite-q solutions

As mentioned in the main text, for TI and TIF models, the differences between the scaling collapse of the finite-q numerics and the infinite-q solution (Fig. 2), can be due to (i) a genuinely different scaling function for finite-q, or (ii) a slow convergence in t to the same scaling function in infinite-q. To investigate the latter possibility, in Figure S9, we compare the finite -L, -t, infinite-q results for the SFF with the infinie-q scaling functions reached at large L and t, for Floquet, TI and TI Floquet models. we find that finite t corrections decay very slowly for TI systems compared to the Floquet systems.

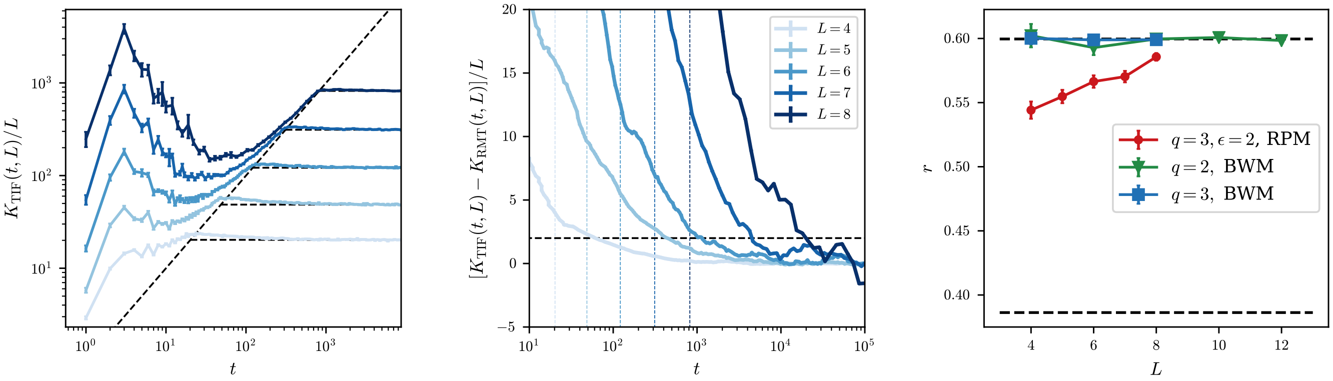

(a) (b) (c)

FIG. S8. **SFF of TI Floquet MBQC circuits.** (a): $K_{\mathrm{TIF}}(t,L)/L$ vs. t for TIF-RPM with $q = 3, \epsilon = 2$, for $L = 4, 5, 6, 7, 8$ (from light to dark blue), and the dashed lines denote the corresponding RMT behavior defined in Eq. SF.4. (b): $K_{\mathrm{TIF}}(t,L)/L - K_{\mathrm{RMT}}(t,L)/L$ vs. t where the vertical lines correspond to t_{Hei} for the L corresponding to the same color. The intersection points with a horizontal line near zero give an estimate of t_{Th} which appear larger than t_{Hei}. (c): Nearest neighbour level spacing ratio r in the zero momentum sector for the three TIF models studied in this paper.

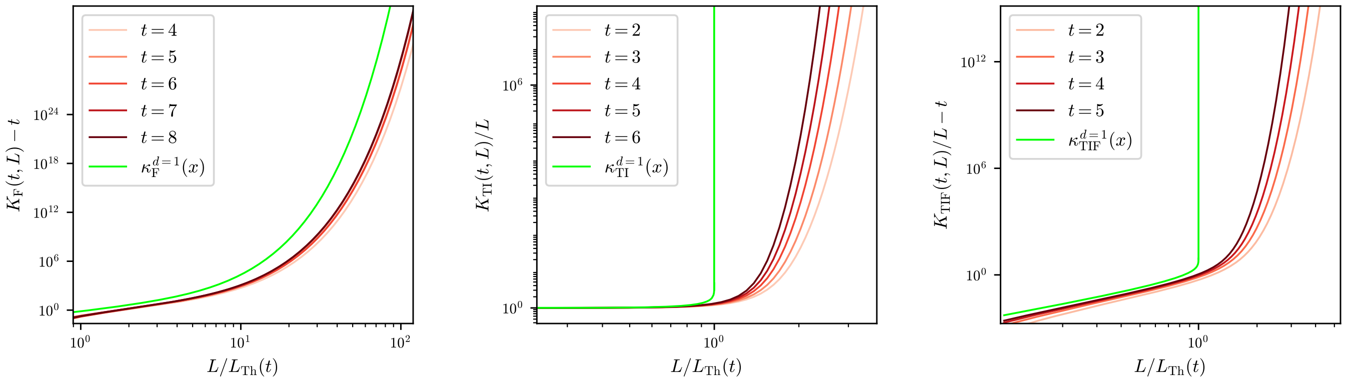

(a) (b) (c)

FIG. S9. **The dependence of infinite-q analytical SFF scaling functions on t and L.** For all three plots, the green curves are the infinite-t, infinite-L, infinite-q scaling functions given in Eq. 13, 10, and 16 (from left to right respectively). (a): $K_{\mathrm{F}}(t,L) - t$ vs $L/L_{\mathrm{Th}}(t)$ for different t where the red curves denote the finite-t, finite-L, infinite-q expression for $K_{\mathrm{F}}(t,L) - t$ (Eq. SB.3), with L_{Th} for F-RPM (from Figure S7). (b): $K_{\mathrm{TI}}(t,L)/L$ vs L/L_{Th} different t, alongside the finite-t, finite-L, infinite-q expression for $K_{\mathrm{TI}}(t,L)/L$ (Eq. SA.5) in reds, with L_{Th} for TI-BWM with $q = 2$. (c): $K_{\mathrm{TIF}}(t,L)/L - t$ vs L/L_{Th} different t, alongside the finite-t, finite-L, infinite-q expression for $K_{\mathrm{TIF}}(t,L)/L - t$ (Eq. SC.2) in reds, with L_{Th} for TI-BWM with $q = 3$.
